# Phase Diagram of 2D Poly(Ethylene Oxide)-*block*-Poly(Propylene Oxide)-*block*-Poly(Ethylene Oxide)—Poly(Dimethylsiloxane) Blends: A Combined Neutron Reflectometry and Sum Frequency Generation Study

**DOI:** 10.1021/acs.langmuir.5c06510

**Published:** 2026-03-16

**Authors:** Aurély Araminthe, Alae El Haitami, Bence Kővágó, Fabrice Cousin, Pablo Sanchez-Puga, John Robert Peter Webster, Philipp Gutfreund, Ellen H. G. Backus, Sophie Cantin

**Affiliations:** † 27004 CY Cergy Paris Université, LPPI, F95000 Cergy, France; ‡ Institute of Physical Chemistry, Faculty of Chemistry, 27258 University of Vienna, Währinger Strasse 42, 1090 Vienna, Austria; § University of Vienna, Vienna Doctoral School in Chemistry (DoSChem), Währinger Strasse 42, 1090 Vienna, Austria; ∥ Laboratoire Léon Brillouin, Université Paris-Saclay, CEA-CNRS UMR 12, F-91191 Gif-sur-Yvette, France; ⊥ 56053 Institut Laue-Langevin, 71 Avenue des Martyrs, 38000 Grenoble, France; # ISIS Neutron and Muon Source, Rutherford Appleton Lab, STFC, Didcot OX11 0QX, England

## Abstract

Polymer blends in thin films offer versatile opportunities for tailoring material properties. Among available deposition methods, the Langmuir film technique enables the formation of well-controlled nanometer-thick films at the air–water interface, revealing unique miscibility and phase behaviors not observed in bulk. In this work, we investigate 2D polymer blends composed of amphiphilic PEO_11_–PPO_35_–PEO_11_ and hydrophobic PDMS using a multiscale approach that combines surface pressure–area isotherms, Brewster angle microscopy (BAM), neutron reflectometry (NR), and sum-frequency generation (SFG) spectroscopy. This methodology enabled us to construct a 2D surface pressure–composition phase diagram, identifying miscibility regions and two distinct first-order phase transitions. Across all compositions, no lateral phase separation was observed at the mesoscopic scale by BAM, even during phase transitions. Instead, NR revealed vertical segregation at specific surface pressures and compositions. In PEO_11_–PPO_35_–PEO_11_-rich blends, a homogeneous monolayer undergoes a transition to a bilayer, with hydrophobic PDMS positioned atop PEO_11_–PPO_35_–PEO_11_. PDMS-rich blends display a similar bilayer structure at all pressures, with the phase transition mainly involving thickening of the PDMS layer, as seen in pure PDMS films. SFG spectroscopy reveals that both polymers have distinct behavior in the blends and in pure films, due to either lateral or vertical interactions. Notably, despite their contrasting hydrophobicities, the polymers exhibit miscibility over a range of compositions and surface pressures, with evidence of attractive interactions. These findings underscore the unique behavior of confined polymer blends and the importance of 2D-specific phase diagrams for designing functional interfacial materials.

## Graphical abstract

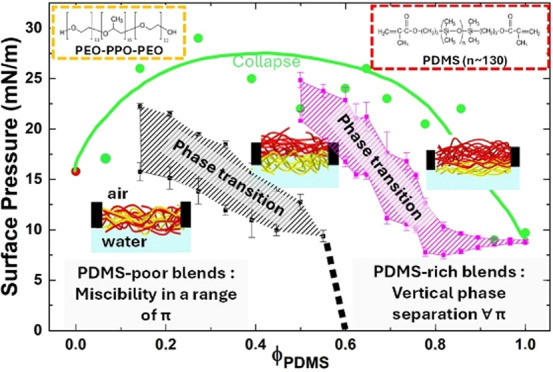

## 1 Introduction

Polymer blends have attracted considerable attention for their potential to combine diverse properties, leading to a range of applications, particularly in the biomedical and industrial sectors.^1^ These blends offer a cost-effective approach to enhance the performance and versatility of pure polymers, provided that compatibility, i.e., miscibility or microscopic scale phase separation, is achieved.^2^ Polymer blends in thin films also present exciting potential for a variety of applications across research fields such as organic electronics, biotechnology, and protective coatings.^3–5^ Compared to chemically heterogeneous block copolymers, the ability to easily modify the chemical composition of films using blends of commercially available polymers offers a flexible, accessible, and cost-effective means of exploring and designing films with specific properties.^5–7^ Nanoconfined polymer blends in thin films are more complex than bulk materials due to the interactions at the substrate/film and film/air interfaces, as well as changes in polymer chain conformation and dynamics.^8^ These effects can alter key physicochemical properties, such as glass transition temperature, crystallization behavior, and miscibility compared to their 3D counterparts. For most thin films made from polymer blends, phase separation is a common occurrence. This phenomenon is typically detected using conventional microscopy techniques. Recent advances in atomic force microscopy, combined with infrared spectroscopy, have also proven to be effective means of revealing phase-separated structures, particularly when the two polymers are chemically similar.^9,10^ In the usual case of phase separation, controlling the morphology is of primary importance to combine or improve the properties of the pure polymers.^6,7,11,12^ The method used to prepare thin films of polymer blends, along with deposition parameters plays a significant role in achieving the desired morphology.^13,14^ For example, conventional dip-coating and spin-coating deposition methods for creating polymer blend thin films highlight the significance of factors like the solvent choice, the withdrawal rate during dip-coating, and the spin speed during spin-coating to control the morphology.^6,15–19^ Additionally, the chemical nature of the substrate must be carefully selected to avoid commonly observed issues such as dewetting or vertical phase separations, which can arise from the differing affinities of both polymers for the substrate and the air.^20–22^ Post-treatments, cross-linking reactions, surface patterning or addition of nanoparticles, have been used to stabilize or adjust the morphology.^23–25^ Despite significant advances in this field, much remains to be understood regarding the fundamental mechanisms governing the miscibility and phase behavior of polymer blends confined in thin films.^15,26^ To address these fundamental questions and achieve a more systematic understanding, experimental approaches that allow precise control over interfacial structure are required. One of the most promising techniques for producing ultrathin, well-structured films of polymer blends is the Langmuir film method. This technique allows for the creation of nanometer-thick films at the air–water interface, offering a high degree of order and precise control over surface density, unlike other deposition methods like spin-coating or dip-coating. It presents a significant opportunity to investigate the behavior of polymer blends in 2D and to understand how composition and thermodynamic parameters influence phase separation and morphology. Several studies of polymer blends at the air–water interface have revealed intriguing phenomena that differ from those observed in bulk 3D materials or exhibit unexpected behavior. For instance, while certain polymer pairs, such as cellulose acetate butyrate/poly­(dimethylsiloxane) (PDMS), show miscibility at the air–water interface for certain compositions, they tend to phase-separate in bulk.^27^ Isotactic poly­(methyl methacrylate) (PMMA) causes phase separation in PMMA/poly­(vinyl cinnamate) blends at the air–water interface, while miscibility occurs in 3D.^28^ Despite their significantly different hydrophobicities, cellulose acetate and polybutadiene are miscible at low surface pressure, while nitrile butadiene rubber and poly­(ethylene oxide)-*block*-poly­(propylene oxide)-*block*-poly­(ethylene oxide) (PEO–PPO–PEO), with similar solubility parameters, are immiscible across all compositions.^29,30^ Furthermore, flexible poly­(vinyl acetate) is miscible with rigid, rod-like poly­(*n*-hexyl isocyanate) or poly­(4-hydroxystyrene) at the air–water interface, but remains immiscible with PDMS or PMMA.^31^ These unexpected results highlight the complexity of polymer blend behavior in 2D and the challenges in predicting miscibility based on 3D systems. In most of these studies, miscibility was inferred from surface pressure–area isotherms, which provide insights into surface excess area and Gibbs free energy.^28,31^ Additional morphological characterization at the air–water interface was sometimes performed by Brewster angle microscopy (BAM) and fluorescence microscopy, or *ex situ* after transfer from the air–water interface to a solid substrate, by atomic force microscopy (AFM), although polymer rearrangement during transfer cannot be excluded.^32–35^ We also demonstrated that coupling BAM with neutron reflectometry (NR) is particularly well-suited for achieving in-depth lateral and vertical morphology analysis, providing a better understanding of phase separation in 2D polymer blends.^31,33,36^ While the miscibility properties of polymer blends are typically studied in 3D through pressure–composition or temperature–composition phase diagrams, which show binodal and spinodal lines and associated blend morphologies, there is still limited research on determining similar phase diagrams for 2D systems.^33^ Yet, the peculiar and often unexpected behavior of polymer blends at the air–water interface clearly shows that conventional 3D models are insufficient to predict 2D miscibility. A deeper and more systematic understanding of phase separation in two dimensions is therefore essential to bridge this knowledge gap.

Addressing this challenge requires complementary characterization techniques capable of probing different length scales and interactions simultaneously. In this work, we studied 2D polymer blends spread at the air–water interface by combining, for the first time to our knowledge, an analysis of film compressibility derived from macroscopic surface pressure–area isotherms, in-plane and out-of-plane morphology determination using BAM and NR, and molecular-scale characterization of polymer–polymer and polymer–water interactions via sum-frequency generation (SFG) spectroscopy. This multiscale approach enabled us to construct a 2D surface pressure–composition phase diagram, thereby identifying regions of miscibility and phase separation together with their corresponding film morphologies. For this purpose, we selected two polymers commonly used as coatings for various applications: (1) an amphiphilic block copolymer of PEO–PPO–PEO with relatively short PEO blocks ((EO)_11_-(PO)_35_-(EO)_11_), hereafter referred to as PEO_11_–PPO_35_–PEO_11_, and (2) hydrophobic PDMS (molar mass ∼ 10,000 g/mol), see Figure 1A.

**Figure 1:**
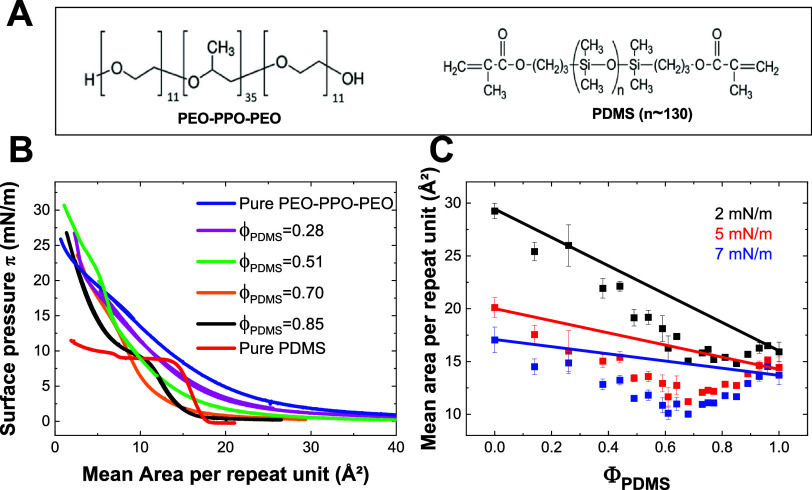
(A) Chemical structures of the polymers. (B) Surface pressure vs mean area per repeat unit isotherms for PEO_11_–PPO_35_–PEO_11_, PDMS, and four PEO_11_–PPO_35_–PEO_11_/PDMS Langmuir films with Φ_PDMS_ = 0.28; 0.51; 0.70 and 0.85. (C) Evolution of the mean area per repeat unit measured for the mixed films as a function of Φ_PDMS_, at 2 mN/m (black), 5 mN/m (red), and 7 mN/m (blue), compared to the additivity law (solid lines).

## 2 Experimental Section

### 2.1 Materials

The triblock copolymer poly­(ethylene oxide)-*block*-poly­(propylene oxide)-*block*-poly­(ethylene oxide) α,ω-bis­(hydroxy)-terminated (PEO_11_–PPO_35_–PEO_11_, supplier reference P18101, *M_n_* 500–2000–500 g/mol) was purchased from PolymerSource. Its deuterated analog, poly­(deuterated ethylene oxide-*b*-deuterated propylene oxide-*b*-deuterated ethylene oxide) α,ω-bis­(hydroxy)-terminated (dPEO_10_–dPPO_34_–dPEO_10_, Sample #P18094, *M_n_* 500–2100–500 g/mol) was also obtained from PolymerSource. The PEO–PPO–PEO used in this study are insoluble in water due to their sufficiently long PPO block and short PEO blocks, which makes them suitable for studying Langmuir films. The poly­(dimethylsiloxane), methacryloxypropyl terminated (PDMS, CAS 58130–03–3, *M_n_* ≈ 10,000 g/mol) was supplied by ABCR (Gelest Inc.). Deuterated poly­(dimethylsiloxane) (dPDMS, *M_n_* ≈ 3000 g/mol) was kindly provided by B. Deloche (Laboratoire de Physique des Solides, UMR 8502, Orsay, France). Heavy water (D_2_O, 99.9% purity) was purchased from Fisher Scientific. All polymers were used as received without further purification. Dichloromethane (HPLC grade, Honeywell) was used as the spreading solvent for all Langmuir film preparations.

### 2.2 Langmuir Film Preparation and Surface Pressure—Area Isotherms

Langmuir films were prepared and studied using a KSV NIMA Medium Langmuir Trough (Biolin Scientific, dimensions 364 × 75 mm) with symmetrical moving barriers. The trough was temperature-controlled at 20.0 ± 0.5 °C through a thermostatic bath circulating coolant beneath the trough, complemented by laboratory air conditioning maintained at 20 °C. The subphase was ultrapure water (resistivity ≥ 18.2 MΩ·cm, Milli-Q) or D_2_O or H_2_O/D_2_O mixtures for neutron reflectometry experiments. Solutions of each polymer (PEO_11_–PPO_35_–PEO_11_, dPEO_10_–dPPO_34_–dPEO_10_, PDMS, dPDMS) were prepared in dichloromethane with concentration in the range of 0.1–0.7 g/L. It was checked that hydrogenated and deuterated versions of each polymer lead to similar isotherms. Binary mixtures were prepared by mixing appropriate volumes of the stock solutions to achieve the desired molar (*x*_PDMS_) or volume (Φ_PDMS_) fraction of PDMS. The molar fraction *x*_PDMS_ is defined as the mole fraction of PDMS repeat units. For PDMS, the repeat unit is [–Si­(CH_3_)_2_ O–] with a molar mass of ∼74 g/mol. For the PEO_11_–PPO_35_–PEO_11_ triblock copolymer, an average repeat unit mass representative of the entire chain was used, calculated from the weighted composition of the copolymer (22 EO units, ∼44 g/mol each, and 35 PO units, ∼58 g/mol each). The molar fraction *x*_PDMS_ is then defined as the ratio of moles of PDMS repeat units to the total moles of polymer repeat units. The solutions were carefully spread onto the water subphase using a Hamilton microsyringe. The solvent was allowed to evaporate for 10 min before compression. Surface pressure–mean area per repeat unit (π–A) isotherms were recorded at a constant compression speed of 6 mm/min. At least three independent isotherms were recorded for each system to ensure reproducibility.

To analyze the isothermal compressibility *C*_s_–π curves derived from the isotherms and to determine the onset and completion of the identified first-order phase transitions, tangents were drawn to the curves at compression stages just before, during, and after the transition. Linear fits were applied to the experimental data in each of these three regions, using the surface pressure ranges over which the response is linear. The intersections of these fitted segments define the onset and completion surface pressures. The corresponding error bars were determined as the standard deviation of measurements obtained on at least three independent *C*_s_–π curves recorded on different films.

### 2.3 Brewster Angle Microscopy

The Langmuir films were visualized *in situ* using an Accurion UltraBAM microscope (Göttingen, Germany). The instrument is equipped with a 50 mW laser diode emitting p-polarized light at 658 nm and a 10× magnification objective providing a lateral resolution of ∼2 μm. For each experiment, the incident Brewster angle for the pure air–water interface was set prior to measurement by performing an angular scan across a range of 52.00° to 54.00° to identify the angle of minimum reflectivity (∼53°). The BAM experiments were conducted in a large Langmuir trough (KSV Nima model, dimensions 580 × 145 mm, allowing for a compression ratio of 18), specifically designed for these observations. The BAM images, acquired with a high-performance CCD camera covering an observation field of 720 × 400 μm^2^, were recorded simultaneously with the compression isotherms, providing a direct correlation between the surface pressure–area data and the morphological evolution of the film. The scale bar is shown directly on the images. Image processing, including automatic background compensation and geometric correction for unskewed images, was performed using the dedicated Image Analysis software provided by Accurion.

### 2.4 Neutron Reflectometry

Neutron reflectometry measurements were performed on two time-of-flight reflectometers: the FIGARO instrument at the Institut Laue-Langevin (ILL, Grenoble, France) and the INTER reflectometer at the ISIS Neutron and Muon Source (U.K.).^37,38^ By measuring the reflectivity at two angles (0.7° and 3.9° on FIGARO, 0.8° and 2.3° on INTER), a range of the scattering vector Q from 0.005 to 0.3 Å^–1^ was assessed. The polymer films were prepared in sealed Langmuir troughs equipped with silicon or quartz windows to minimize atmospheric contamination and prevent H/D exchange. Throughout the NR measurements at constant surface pressure, all films remained thermodynamically stable (constant mean area per repeat unit). The contrast variation method was employed using either dPEO_10_–dPPO_34_–dPEO_10_/PDMS or PEO_11_–PPO_35_–PEO_11_/dPDMS film and various H_2_O/D_2_O mixtures as subphases (Table 1). The measured reflectivity curves were processed by subtracting incoherent scattering and background. Data analysis was performed using the Motofit software.^39^ Data from multiple contrast conditions were fitted simultaneously to ensure model robustness. The fitting procedure began with a single-layer model, with additional layers introduced when the fit quality was not good. The SLD, thickness, roughness, and water content were deduced for each layer. They correspond to the fit with the lowest χ^2^ value, with estimated uncertainties of ±0.1 × 10^–6^ Å^–2^ for SLD, ±1–2 Å for thickness, ±1–2 Å for interfacial roughness, and ±10% for water content. The following parameter bounds were applied during fitting: layer thickness (0–100 Å), scattering length density (−0.56 to 6.73 × 10^–6^ Å^–2^), interfacial roughness (2–10 Å), and water fraction (0–100%). To assess the reliability of the layer parameters derived from the NR data, the mass *m* of each polymer calculated from the polymer volume fraction-depth profile was compared to the mass *m*_*S*_ of polymer spread at the air–water interface. The polymer mass *m* is given by

$$m=d_{e}\times S\times \rho$$

where *d*_e_, the effective thickness of the polymer layer, corresponds to a water-free polymer layer. *d*_e_ is calculated by integrating the polymer volume fraction over the entire depth *z*; *S* is the surface area covered by the film at the specific surface pressure, and ρ is the polymer density. The relative deviation Δ*m* on the polymer mass, defined as Δ*m* = (*m – m_S_*) /*m_S_*, is reported in Table SI-1.

**Table 1:** Scattering Length Densities (SLD) of Polymers, Studied Blends, and Different H_2_O/D_2_O Subphases Used in NR^a^

system	SLD (×10^–6^ Å^–2^)	Φ_D2O_ (%) in H_2_O/D_2_O subphase contrast-matched to corresponding material
H_2_O	–0.56	0.0
D_2_O	6.35	100.0
dPDMS	5.14	82.5
dPEO_10_-dPPO_34_-dPEO_10_	6.73	100.0
PDMS	0.08	9.32
PEO_11_–PPO_35_–PEO_11_	0.43	14.2
Φ_PDMS_ = 0.28 (*x*_PDMS_ = 0.2): dPEO_10_–dPPO_34_–dPEO_10_/PDMS	4.87	78.6
Φ_PDMS_ = 0.28 (*x*_PDMS_ = 0.2): PEO_11_–PPO_35_–PEO_11_/dPDMS	1.69	32.5
Φ_PDMS_ = 0.51 (*x*_PDMS_ = 0.4): dPEO_10_–dPPO_34_–dPEO_10_/PDMS	3.35	56.6
Φ_PDMS_ = 0.51 (*x*_PDMS_ = 0.4): PEO_11_–PPO_35_–PEO_11_/dPDMS	2.76	48.0
Φ_PDMS_ = 0.70 (*x*_PDMS_ = 0.6): dPEO_10_–dPPO_34_–dPEO_10_/PDMS	2.08	38.2
Φ_PDMS_ = 0.70 (*x*_PDMS_ = 0.6): PEO_11_–PPO_35_–PEO_11_/dPDMS	3.7	61.6
Φ_PDMS_ = 0.85 (*x*_PDMS_ = 0.8): PEO_11_–PPO_35_–PEO_11_/dPDMS	4.45	72.2

a Φ and *x* represent the volume and molar fractions, respectively.

### 2.5 Sum Frequency Generation

SFG experiments were performed using a broad-band SFG spectrometer using the phase resolved SFG setup described in ref 40. For standard SFG experiments the local oscillator was removed from the setup. The Langmuir films were prepared in microtrough G1 (Kibron, Finland). As compressing the monolayer, SFG spectra were collected at fixed surface pressure in the C–H/O-H stretching region (2750–3600 cm^–1^), both in the SSP (s-polarized SFG, s-polarized visible, p-polarized IR) and SPS polarization combinations. Each spectrum was averaged for 5 min, background subtracted, and normalized against a reference spectrum from a *z*-cut quartz crystal to account for the IR intensity profile and laser fluctuations. The spectra were fitted using a Lorentzian model to extract the amplitude, frequency, and width of the vibrational resonances (see for example ref 41). The relevance of the fitting parameters, and especially the sign of the amplitudes, was checked by comparing the real and imaginary parts of the nonlinear susceptibility extracted from the fit and measured using a phase-resolved SFG setup. For these selected experiments, a round Teflon trough with a diameter of 8 cm was used.

To account for the different amounts of each polymer in the blends and in the pure films, the SFG amplitudes (Amplitude_norm_) of specific vibrational bands in the blends were normalized according to the following equation

$$\mathrm{Amplitud}e_{\mathrm{norm}}=\mathrm{Amplitud}e_{\mathrm{blend}}\times \left[A_{\mathrm{mix}}/\left(A_{\mathrm{pure}}\times x\right)\right]$$

where Amplitude_blend_ is the raw amplitude, *A*_mix_ and *A*_pure_ are the mean areas per repeat unit of the blend and the pure polymer film, respectively, at the given surface pressure, and *x* is the mole fraction of the polymer in the blend. Therefore, any change in Amplitude_norm_ compared with the pure film is attributed solely to changes in molecular orientation.

## 3 Results and Discussion

### 3.1 Phase Diagram and Lateral Morphology

The thermodynamic properties of pure PDMS, pure PEO_11_–PPO_35_–PEO_11_, and their blends with varying volume fractions of PDMS (Φ_PDMS_, the corresponding molar fraction x_PDMS_ is also provided in Table 1) were investigated using compression isotherms at the air–water interface, complemented by Brewster angle microscopy (BAM) observations. The isotherm of the pure PDMS film (Figure 1B, red trace) exhibits a steep, linear rise in surface pressure below a mean area per repeat unit of approximately 18 Å^2^—a value consistent with closely packed PDMS monolayers at the air–water interface prior to chain folding—reaching a plateau at 9.2 mN/m.^42^ Below this plateau indicative of a first-order phase transition, the film is in the so-called semidilute regime, in which polymer chains are in contact and progressively interpenetrate as the surface pressure increases.^43^ BAM images reveal a homogeneous film, consistent with a uniform dense state. The transition at 9.2 mN/m has been attributed to the progressive horizontal folding of PDMS chains as the mean area per repeat unit decreases along the plateau.^44^ However, no visual indication of this phase transition is observed in BAM, likely due to the refractive index of PDMS being close to that of water.^45^ Beyond the plateau, the surface pressure increases slightly to around 10 mN/m, marking the onset of monolayer collapse associated with the appearance of three-dimensional structures as observed by BAM. In contrast, the pure PEO_11_–PPO_35_–PEO_11_ Langmuir film (Figure 1B, blue trace) displays a gradual and continuous rise in surface pressure starting from 45 Å^2^ per repeat unit, until collapse at approximately 16 mN/m. As expected from the short PEO blocks, the film remains in the semidilute regime, adopting the characteristic “pancake” conformation of block copolymers at the air–water interface.^46,47^ BAM analysis confirms a homogeneous film in this dense state up to the point of collapse.

Figure 1B presents representative isotherms for the mixed PDMS/PEO_11_–PPO_35_–PEO_11_ films at selected compositions, but additional isotherms were measured to establish the phase diagram shown in Figure 2. Two distinct behaviors are observed depending on the composition. For PDMS-rich mixtures (Φ_PDMS_ > 0.70), a surface pressure pseudoplateau appears in the range of 7–10 mN/m, indicative of a first-order phase transition within a mixture. Unlike the sharp and well-defined plateau of pure PDMS, this phase transition occurs over a broader range of surface pressures (a few mN/m). It is followed by a steep rise in surface pressure, reaching values above 25 mN/m– exceeding those observed for pure PEO_11_–PPO_35_–PEO_11_. As Φ_PDMS_ decreases within these PDMS-rich blends, the pseudoplateau shortens and the surface pressure range over which the phase transition occurs broadens. For instance, the transition spans from 7.7 to 9.6 mN/m at Φ_PDMS_ = 0.85 while it extends from 10.1 to 15.4 mN/m at Φ_PDMS_ = 0.75. This dependence on blend composition suggests molecular-level interactions between PDMS and PEO_11_–PPO_35_–PEO_11_ at the air–water interface. For Φ_PDMS_ = 0.70, the isotherm no longer displays a clear pseudoplateau but only a weak inflection between 10 and 15 mN/m. For blends with lower PDMS volume fraction (Φ_PDMS_ < 0.70), the surface pressure increases continuously up to film collapse, closely resembling the isotherm of pure PEO_11_–PPO_35_–PEO_11_.

**Figure 2:**
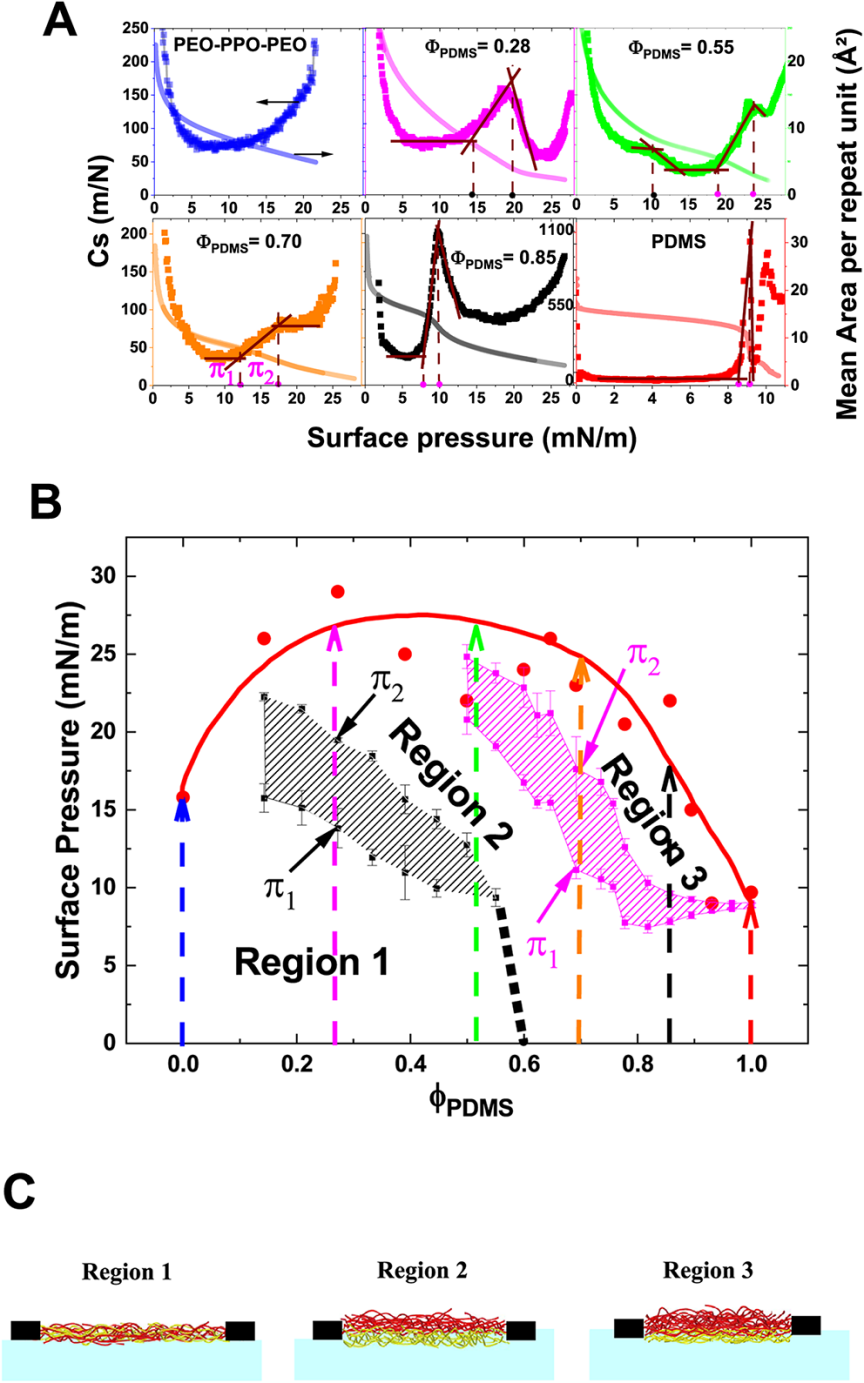
(A) Compressibility *C*_s_ as a function of surface pressure π, derived from π–A isotherms for PEO_11_–PPO_35_–PEO_11_ and PDMS Langmuir films, as well as certain blends (dots). The isotherms are presented using a dual-axis format (solid lines). (B) Surface pressure π–composition Φ_PDMS_ phase diagram of PEO_11_–PPO_35_–PEO_11_/PDMS blends at the air–water interface. The gray and pink hatched regions indicate the domains where two 1st-order phase transitions occur. Red points represent the film collapse pressures determined by BAM. The four arrows indicate the blend compositions selected for SFG and NR analyses: magenta for Φ_PDMS_ = 0.28, green for Φ_PDMS_ = 0.51, orange for Φ_PDMS_ = 0.70, and black for Φ_PDMS_ = 0.85. The dotted line at around Φ_PDMS_ = 0.6 indicates the extension of the phase transition for PEO_11_–PPO_35_–PEO_11_-rich blends to zero surface pressure, as discussed in Section 3.4. (C) Schematics of the vertical film structure deduced from NR measurements in regions 1, 2, and 3. The red and yellow polymer chains represent PDMS and PEO_11_–PPO_35_–PEO_11_, respectively.

To further investigate the nature of the interactions between the two polymers at the air–water interface, the mean area per repeat unit (*A*) was plotted as a function of Φ_PDMS_ at fixed surface pressures of 2, 5, and 7 mN/m—below the plateau observed for pure PDMS. Experimental values were then compared with those predicted by the additivity rule, which describes either ideal mixing or complete phase separation

$$A=\Phi_{\mathrm{PDMS}}\cdot A_{\mathrm{PDMS}}+\left(1-\Phi_{\mathrm{PDMS}}\right)\cdot A_{{\mathrm{PEO}}_{11}-{\mathrm{PPO}}_{35}-{\mathrm{PEO}}_{11}}$$

The results presented in Figure 1C show a systematic negative relative deviation from the additivity line across all compositions, with the largest deviation occurring at Φ_PDMS_ around 0.7. This deviation could be indicative of miscibility at these surface pressures, as it reflects attractive interactions between PDMS and PEO_11_–PPO_35_–PEO_11_ at the interface—a contraction of the film relative to the ideal blend.

To more precisely characterize phase transitions in polymer blends, the evolution with the surface pressure of isothermal compressibility *C*_s_, $C_{s}=-\frac{1}{A}{\left(\frac{dA}{d\pi }\right)}_{T}$ , was calculated from the compression isotherms for all studied Φ_PDMS_ fractions. The *C*_s_–π curves provide a sensitive means of detecting phase transitions. First-order phase transitions appear as a sharp increase in *C*_s_, consistent with the presence of a surface pressure plateau in the isotherm. In contrast, second-order phase transitions, which typically correspond to a kink in the isotherm, are identified by a distinct change in slope, reflecting a transition to a new phase with lower *C*_s_. Figure 2A displays the *C*_s_–π curves for the pure polymer films and the four representative blends, with their respective isotherms plotted on a dual axis. During compression at zero surface pressure, all curves initially exhibit a decrease in *C*_s_ until a homogeneous dense state is reached near the lift-off point of the isotherm. Subsequently, first- or second-order phase transitions may occur at nonzero surface pressures, and at high surface pressure, a sharp rise in *C*_s_ signals the collapse of the monolayer. The *C*_s_–π curves in Figure 2A highlight several distinct behaviors. First, after the initial decrease, a sharp increase in *C*_s_, characteristic of a first-order phase transition, is observed for pure PDMS (starting around 8.5 mN/m) as well as for PDMS-rich blends, as shown for Φ_PDMS_= 0.70 (starting around 11 mN/m) and 0.85 (starting around 8 mN/m). To precisely identify the surface pressure range over which these transitions occur, tangents were drawn (See Section 2.2) to the *C*_s_–π curves at compression steps just before, during, and after the transition (Figure 2A). The intersection points of these tangents define the onset (π_1_) and completion (π_2_) surface pressures of the transition, as illustrated for Φ_PDMS_ = 0.70 in Figure 2A. These values were then compiled into a surface pressure versus composition phase diagram, π = *f*(Φ_PDMS_), shown in Figure 2B. Owing to the sensitivity of C_s_ analysis to subtle changes in slope, this phase transition—represented by the pink hatched region—is clearly detected for PDMS-rich blends with Φ_PDMS_ ≥ 0.51. With decreasing PDMS volume fraction, the onset surface pressure π_1_ increases continuously, while the pressure range over which the transition occurs (π_2_–π_1_) broadens. This behavior strongly suggests the presence of specific interactions between the two polymers within the blend. Interestingly, for PEO_11_–PPO_35_–PEO_11_-rich blends, a first-order transition is detected in the compressibility curve, although the C_s_-π curve of the pure PEO_11_–PPO_35_–PEO_11_ film shows no sign of a phase transition. This transition, not clearly visible in the raw isotherms, is illustrated in Figure 2A for Φ_PDMS_ = 0.28, where the phase transition is observed to start at π_1_ = 13.8 mN/m and end at π_2_ = 19.5 mN/m. Applying the same analysis across all PEO_11_–PPO_35_–PEO_11_-rich blends reveals similar behavior within the range 0.14 ≤ Φ_PDMS_ < 0.51. The corresponding transition points are plotted in black on the phase diagram in Figure 2B, delineating the gray hatched region. As Φ_PDMS_ increases, the onset surface pressure π_1_ progressively decreases, and the surface pressure range of the phase transition, π_2_ – π_1_, narrows. At Φ_PDMS_ = 0.51 and 0.55, only a subtle break in the slope of the *C*_s_–π curve is observed around 10 mN/m, which may be indicative of a second-order phase transition (Figure 2A). Notably, for Φ_PDMS_ = 0.51 and 0.55, two distinct phase transitions are detected during compression, suggesting the existence of two separate phenomena. This observation supports the interpretation that the two phase transitions mapped on the π = f­(Φ_PDMS_) phase diagram correspond to different mechanisms. Accordingly, they will be referred to as the *phase transition in PEO_11_*–*PPO_35_*–*PEO_11_-rich films* (gray hatched region) and the *phase transition in PDMS-rich films* (pink hatched region).

To better analyze the thermodynamic behavior of the films, we measured the isotherms of certain blends (Φ_PDMS_ = 0.51 and 0.85) during two successive cycles of compression–expansion experiments, either up to 5 mN/m (below the phase transition) or to a surface pressure above the phase transition (15 mN/m for Φ_PDMS_ = 0.51, 12 mN/m for Φ_PDMS_ = 0.85) (Figure SI-1). It is worth noting that in all cases, the hysteresis between compression and expansion is negligible, and the second cycle consistently overlaps with the first within experimental uncertainties. These experiments demonstrate that both phase transitions are reversible.

All PEO_11_–PPO_35_–PEO_11_/PDMS blend films were analyzed using BAM (Figure SI-2). Across all volume fractions Φ_PDMS_ and throughout the entire surface pressure range up to collapse, the films appeared homogeneous. This indicates the absence of lateral phase separation or phase coexistence at the mesoscopic scale, even during the two identified phase transitions. Film collapse was eventually observed with the formation of 3D aggregates. The corresponding collapse pressures are indicated by red dots in Figure 2B. Notably, for all blended films, collapse occurred at surface pressures significantly higher than those of the pure polymer films, suggesting the presence of intermolecular interactions between PDMS and PEO_11_–PPO_35_–PEO_11_ at the air–water interface.

In summary, detailed analysis of the compressibility curves revealed two first-order phase transitions that remained undetectable by BAM. To gain further insight into the molecular-scale organization underlying these phase transitions, a complementary approach combining neutron reflectometry (NR) and sum-frequency generation (SFG) spectroscopy was employed.

### 3.2 Methodology for Achieving the Structure and Molecular-Scale Organization of the 2D Polymer Blends

Pure PEO_11_–PPO_35_–PEO_11_ and PDMS, along with their blends, were analyzed at the air–water interface by neutron reflectometry (NR) to investigate their vertical structure and by sum-frequency generation spectroscopy (SFG) to study interactions between both polymers.

#### 3.2.1 Pure Polymers as Reference

##### 3.2.1.1 PEO_11_–PPO_35_–PEO_11_

The structure of deuterated dPEO_10_–dPPO_34_–dPEO_10_ was investigated by NR on two H_2_O/D_2_O subphases. The first subphase was contrast-matched to the hydrogenated PDMS polymer counterpart used in the blends, while the second was chosen with an SLD of 3.10^–6^ Å^–2^ intermediate between that of dPEO_10_–dPPO_34_–dPEO_10_ and PDMS. The NR curves and the corresponding SLD profiles as a function of depth (*z*) profiles, obtained from a simultaneous fit using a single-layer model, are presented in Figure SI-3A for the film at 5 mN/m. From these SLD-depth profiles, the volume fractions of PEO_11_–PPO_35_–PEO_11_ (Φ_PEO_11_–PPO_35_–PEO_11__) and water (Φ_water_) were calculated as a function of depth *z*, as explained in a previous work (Figure SI-3B).^29^ This figure also shows the evolution of these profiles at 10 mN/m, based on the same fitting procedure. The extracted layer parameters are summarized in Table 2. The PEO_11_–PPO_35_–PEO_11_ film consists of a very thin polymer layer, regardless of surface pressure. The thickness, measuring 5.4 Å at 5 mN/m and 5.9 Å at 10 mN/m, remains largely unchanged with surface pressure, while the film’s hydration decreases from 37% to 21% over the same compression range. Both observations are consistent with the absence of a phase transition in the isotherm and the presence of hydrophilic PEO blocks. To assess the reliability of the layer parameters derived from the NR data, the mass *m* of PEO_11_–PPO_35_–PEO_11_ calculated from the polymer volume fraction profile was compared to the mass *m*_*S*_ of PEO_11_–PPO_35_–PEO_11_ spread at the air–water interface (see Section 2.4). The relative deviation Δ*m* on the polymer mass is reported in Table SI-1. The values of around −19 and −27% may seem relatively high, but they can be partly attributed to the extremely thin nature of the layer (∼ 5 Å). For instance, at 5 mN/m, a thickness of 6.6 Å instead of 5.4 Å would reconcile the calculated mass *m* with the expected value, which is within the estimated thickness uncertainty of approximately 1 Å.

**Table 2:** Vertical Structure Derived from Fits of the NR Curves for Pure PEO_11_–PPO_35_–PEO_11_ (Φ_PDMS_ = 0) and Pure PDMS (Φ_PDMS_ = 1) Films, as well as Certain Blends with Volume Fraction Φ_PDMS_ (or Molar Fraction *x*_PDMS_), at Different Surface Pressures^a^

Φ_PDMS_	*x*_PDMS_	surface pressure	vertical structure	layer composition polymer/water fraction	thickness (Å)	σ_up_ (Å)	σ_bot_ (Å)
**0**	**0**	5.0	single layer	PEO_11_–PPO_35_–PEO_11_/37%	5.4	2.0	2.0
		10.0	single layer	PEO_11_–PPO_35_–PEO_11_/21%	5.9	2.5	2.5
**0.28**	**0.2**	5.0	single layer	Homogeneous blend/0%	4.9	2.0	2.0
		23.0	bilayer: upper layer	PDMS/0%	6.0	2.5	3.4
			bottom layer	PEO_11_–PPO_35_–PEO_11_ /72%	20.7	3.4	2.5
**0.51**	**0.4**	5.0	single layer	homogeneous blend/20%	7.8	2.0	2.0
		17.0	bilayer: upper layer	PDMS/0%	6.7	2.0	2.7
			bottom layer	PEO_11_–PPO_35_–PEO_11_/72%	16.2	2.7	2.0
**0.70**	**0.6**	5.0	bilayer: upper layer	PDMS/0%	6.1	2.0	2.0
			bottom layer	PEO_11_–PPO_35_–PEO_11_/21%	3.1	2.0	2.0
		20.0	bilayer: upper layer	PDMS/0%	12.2	2.5	3.2
			bottom layer	PEO_11_–PPO_35_–PEO_11_/37%	7.3	3.2	2.5
**0.85**	**0.8**	5.0	bilayer: upper layer	PDMS/0%	9.8	2.0	2.0
			bottom layer	PEO_11_–PPO_35_–PEO_11_/0%	1.7	2.0	2.0
		15.0	bilayer: upper layer	PDMS/0%	13.9	4.5	4.5
			Bottom layer	PEO_11_–PPO_35_–PEO_11_/0%	3.1	4.5	4.5
**1**	**1**	5.0	single layer	PDMS/0%	7.7	2.1	2.1
		9.2	single layer	PDMS/0%	9.0	2.2	2.2
		A = 14.1 Å^2^					
		9.2	single layer	PDMS/0%	12.6	2.5	2.5
		A = 7.7 Å^2^					

a For each layer, the polymer composition, water fraction, and thickness are provided, along with the roughness values at both interfaces.

The SFG spectra of the PEO_11_–PPO_35_–PEO_11_ film in SSP (Figure 3A) and SPS polarizations (Figure SI-4A) consistently show, at all studied surface pressures, different bands attributed to polymer bond vibrations and a broad band arising from water (Table 3). A band assigned to the CH symmetric stretching in the CH_2_ groups, common to both the PEO and PPO blocks, appears at 2873 cm^–1^ in SSP polarization and at 2865 cm^–1^ in SPS polarization. The corresponding asymmetric CH_2_ stretching vibration is very weak and observed only in SSP polarization at 2908 cm^–1^.^48^ The bands associated with the CH_3_ symmetric and asymmetric stretching vibrations from the PPO block are detected in SSP polarization at 2934 and 2974 cm^–1^, and in SPS polarization, at 2918 and 2967 cm^–1^, respectively. Additionally, a very weak band at 2990 cm^–1^ was required to properly fit the spectra in SPS polarization, although it could not be clearly assigned. These spectral features are consistent with previous results reported for another PEO–PPO–PEO Langmuir film composed of blocks with different lengths.^49^ Finally, the broad water band can be best described with two bands with opposite relative sign centered at 3120 and 3460 cm^–1^ in SSP polarization, and at 3250 and 3560 cm^–1^ in SPS polarization. The strong band at 3560 cm^–1^ in SPS originates from the asymmetric stretch mode. Figures SI-5A,C and SI-6A,C show the real and imaginary parts of the nonlinear susceptibility extracted from the fit and measured at 5 mN/m, which confirm the relevance of the fitting parameters. When increasing the surface pressure from 5 to 15 mN/m, the IR frequencies of all bands remain nearly unchanged. Their amplitude evolution is shown in Figure 3B, while Figure SI-7 reports the evolution of the amplitude ratio of the CH_3_ or CH_2_ vibration between SSP and SPS polarizations (SSP/SPS ratio) on one hand (Figure SI-7A) and between symmetric (s) and asymmetric (as) modes (s/as ratio) in SSP or SPS polarization (Figure SI-7B), allowing for better characterization of the chemical groups’ orientation upon film compression. Considering the CH_3_ groups only in the PPO block, the SSP/SPS ratio slightly increases for the symmetric vibration and slightly decreases for the asymmetric vibration, while the s/as ratio slightly increases in SSP polarization and slightly decreases in SPS polarization. All these evolutions suggest that the CH_3_ dipole moments are oriented more perpendicular to the air–water interface as the surface pressure increases. For the CH_2_ symmetric stretching vibrations, associated with both PPO and PEO blocks, the SSP/SPS ratio increases with the surface pressure, indicating that these groups in average also tend to straighten up.

**Figure 3:**
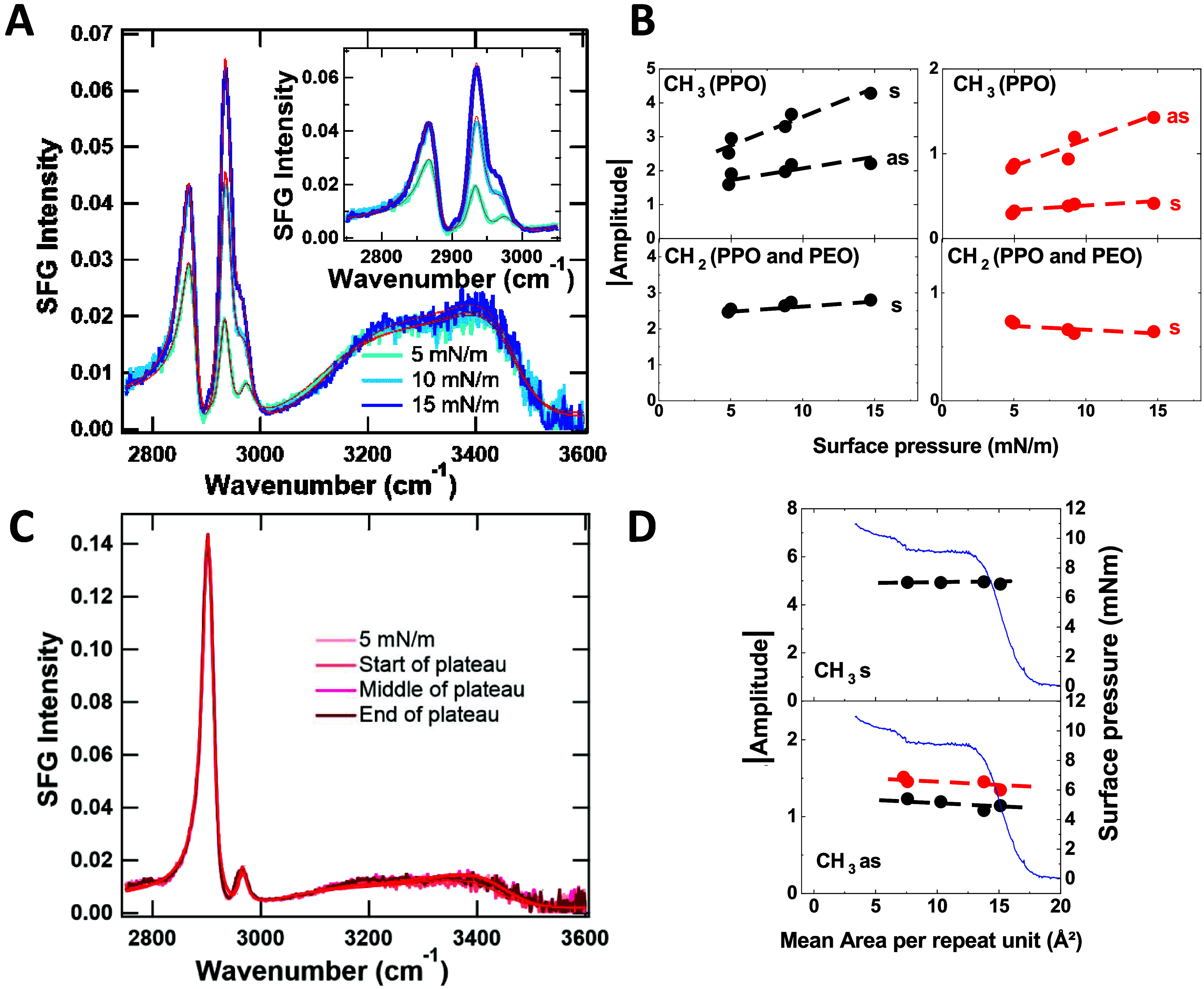
SFG spectra in SSP polarization at different surface pressures in the C–H/O-H vibrational region (2750–3600 cm^–1^) (A, C), and evolution of the absolute value of the amplitude of the relevant SFG bands during compression in SSP (black) and SPS (red) polarizations (B, D) for PEO_11_–PPO_35_–PEO_11_ (A, B) and PDMS (C, D) Langmuir films. The spectra are fitted using a Lorentzian line shape model (solid lines). Band amplitude evolutions are plotted as a function of surface pressure for PEO_11_–PPO_35_–PEO_11_ (B), and as a function of the mean area per repeat unit for PDMS (D). For PDMS, the isotherm is shown using a dual-axis format. Band assignments are indicated, with s and as denoting symmetric and asymmetric stretching vibrations, respectively. Black and red dots indicate bands observed in SSP and SPS polarizations, respectively. Dotted lines are included as visual guides.

**Table 3:** Wavenumber (IR frequency), Bandwidth, and Relative Sign of the SFG Bands Measured in SSP and SPS Polarizations for Pure PEO_11_–PPO_35_–PEO_11_ and PDMS Langmuir Films at 5 mN/m, along with Their Vibrational Assignment (s: Symmetrical Stretching Vibration, as: Asymmetrical Stretching Vibration)^a^

	wavenumber (cm^–1^)/bandwidth (cm^–1^)/relative band sign (values at 5 mN/m)				
Film	SSP	SPS	assignment	bands monitored within the mixed films	references
PDMS	2904/25/+	2900/30/–	CH_3_ s (polymer)	in SSP polarization	^44^
				PDMS-band	
	2966/20/–	2959/15/+	CH_3_ as (polymer)		
		2990/50/–			
	3020/300/–	3220/340/–	O–H (water)	in SSP polarization	^50–52^
	3440/200/+	3510/315/+		OH-band	
PEO_11_–PPO_35_–PEO_11_	2873/30/+	2865/40/+	CH_2_ s (polymer, in both PEO and PPO blocks)	in SSP polarization	^49^
				PEO_11_–PPO_35_–PEO_11_-band 1	
	2908/15/+		CH_2_ as (polymer, in both PEO and PPO blocks)		
	2934/25/+	2918/30/–	CH_3_ s (polymer, PPO block only)	in SSP polarization	
				PEO_11_–PPO_35_–PEO_11_-band 2	
	2974/30/–	2967/20/+	CH_3_ as (polymer, PPO block only)		
		2990/30/–			
	3120/300/–	3250/340/–	O–H (water)	in SSP polarization	^50–52^
	3460/175/+	3560/315/+		OH-band	

a The bands monitored in the mixed films are indicated in the second last column. References for band assignment are indicated in the last column.

##### 3.2.1.2 PDMS

The structure of a PDMS Langmuir film was investigated by NR using both hydrogenated PDMS and deuterated PDMS (dPDMS) deposited on a H_2_O/D_2_O subphase with an SLD of 3.10^–6^ Å^–2^. The NR curves and the corresponding scattering length density (SLD) profiles as a function of depth (z), obtained through a simultaneous fit using a single-layer model, are shown in Figure SI-8A for a film at 5 mN/m. From these profiles, the volume fractions of PDMS (Φ_PDMS_) and water (Φ_water_) were calculated as a function of depth *z* at 5 mN/m and across the compression range (Figure SI-8B). PDMS forms a thin layer with a thickness of 7.7 Å at 5 mN/m, which increases from 9.0 to 12.6 Å along the 9.2 mN/m surface pressure plateau, as the mean area per repeat unit A decreases from ∼14.1 to ∼7.7 Å^2^. The absence of any layer hydration throughout compression is consistent with the high hydrophobicity of PDMS.^32^ The relative deviation Δ*m* in the PDMS mass derived from NR data is presented in Table SI-1. Consistent results (Δ*m* = −6 and −7%) are obtained in the semidilute regime (5 mN/m and early stages of the surface pressure plateau). A higher relative deviation of −26% is observed at the end of the plateau, likely due to the slightly increased roughness attributed to the formation of folded layers, as reported in the literature.^44^

Figures 3C and Figure SI-4B show the SFG spectra of PDMS Langmuir films recorded in SSP and SPS polarizations, respectively. Figures SI-5B,D show the real and imaginary parts of the nonlinear susceptibility extracted from the fit and measured at 5 mN/m. At all surface pressures, the spectra exhibit two bands corresponding to the C–H symmetric and asymmetric stretching vibrations of the CH_3_ methyl groups in the polymer backbone. At 5 mN/m, the CH_3_ symmetric stretching vibration is observed at 2904 cm^–1^ in SSP polarization and at 2900 cm^–1^ in SPS polarization, while the asymmetric stretching appears at 2966 and 2959 cm^–1^ in SSP and SPS polarizations, respectively (Table 3). It is also worth noting that, in SPS polarization, a very weak band at 2990 cm^–1^ had to be included to achieve an accurate fit of the spectra, although its origin remains unassigned. Notably, no sp^2^ C–H stretching vibrations (expected beyond 3000 cm^–1^) related to the C=C–H group of the terminal methacrylate groups are detected, likely due to the large number (∼130) of dimethylsiloxane repeat units in the PDMS chain. As with PEO_11_–PPO_35_–PEO_11_, the broad water band is described with two opposite bands centered in SSP polarization at 3020 cm^–1^ and 3440 cm^–1^, and in SPS polarization at 3220 cm^–1^ and 3510 cm^–1^.^50^ Compared to the O–H stretching vibrations in the presence of the PEO_11_–PPO_35_–PEO_11_ film, a systematic red shift is observed in both polarizations, indicating stronger hydrogen bonding among water molecules when a more hydrophobic polymer like PDMS is spread at the air–water interface (Table 3).^51,52^

Figure 3D presents the evolution of the SFG amplitudes of the PDMS-related vibrational bands as a function of mean area per repeat unit, during compression, from 5 mN/m to the end of the 9.2 mN/m surface pressure plateau. Figure SI-9 shows the SSP/SPS ratio for the CH_3_ asymmetric stretching vibration and the s/as ratio for the CH_3_ in SSP polarization. Notably, in both polarizations, the amplitudes of the symmetric and asymmetric CH_3_ stretching vibrations remain nearly constant throughout compression, particularly across the 9.2 mN/m surface pressure plateau. This is confirmed by the evolution of the ratios shown in Figure SI-9. These findings align with those reported by Kim et al. for a PDMS Langmuir monolayer of comparable molecular weight,^44^ where the constant amplitude was attributed to the formation of folded layers. In addition, the much more intense band for the CH_3_ s compared to the as in SSP, and vice versa in SPS, indicates that the CH_3_ dipoles are oriented more perpendicular to the water surface than parallel.

#### 3.2.2 Mixed Films: Methodology

##### 3.2.2.1 Neutron Reflectometry

For NR, to enhance the scattering length density (SLD) contrast between the polymer blend layer and the subphase—and thereby allow a more accurate structural determination—we used either a deuterated PEO_11_–PPO_35_–PEO_11_ mixed with hydrogenated PDMS, or a hydrogenated PEO_11_–PPO_35_–PEO_11_ mixed with deuterated PDMS. These blends are hereafter referred to as dPEO_10_–dPPO_34_–dPEO_10_/PDMS and PEO_11_–PPO_35_–PEO_11_/dPDMS, respectively. The SLDs of the individual polymers and their blends, as well as those of the H_2_O/D_2_O subphases used, are listed in Table 1. To better assess the miscibility behavior, each polymer blend Langmuir film was first spread on a H_2_O/D_2_O subphase whose SLD was contrast-matched to that of the mixed film, if homogeneous. This approach is justified by BAM observations, which revealed that all mixed films are laterally homogeneous at length scales greater than a few microns, i.e., the neutron coherence length. If the NR curve of the film matches that of the subphase, and assuming the film’s SLD is the weighted average of the individual polymers’ SLD (SLD = Φ_PDMS_ SLD_PDMS_ + (1 – Φ_PDMS_) SLD_PEO_11_–PPO_35_–PEO_11__), this would indicate that the film is homogeneous both laterally and vertically on these length scales.^29,53^

To further analyze the structure, we employed at least two additional H_2_O/D_2_O subphase contrasts for each blend and fitted all corresponding NR curves simultaneously. The simultaneous fit of multiple contrast conditions, using both dPEO_10_–dPPO_34_–dPEO_10_/PDMS and PEO_11_–PPO_35_–PEO_11_/dPDMS films, with subphase SLDs spanning a wide range of values, significantly improves the robustness of the analysis. In addition, the fitting procedure explicitly accounts for the experimental uncertainties: data points with larger error bars naturally carry less weight than those with smaller uncertainties. To guide the choice of the fitting model (single-layer versus multilayer), we primarily rely on measurements performed on the subphase contrast-matched to the homogeneous film. The parameters extracted from the fits – namely the thickness and interfacial roughness of each layer – are summarized in Table 2.

##### 3.2.2.2 SFG

SFG spectra recorded at 5 mN/m in SSP and SPS polarizations for the four studied blends are presented in Figure 4, along with those of the two corresponding pure polymer films. Figure SI-10 shows all the spectra measured for the four studied blends at various surface pressures, together with their corresponding fits. To investigate the behavior of PEO_11_–PPO_35_–PEO_11_ and PDMS in the blends and to explore their interactions, we focused on SFG bands that could be attributed exclusively to either PEO_11_–PPO_35_–PEO_11_ or PDMS, and SFG bands with insufficient intensity were excluded from the analysis (Table 3).

**Figure 4:**
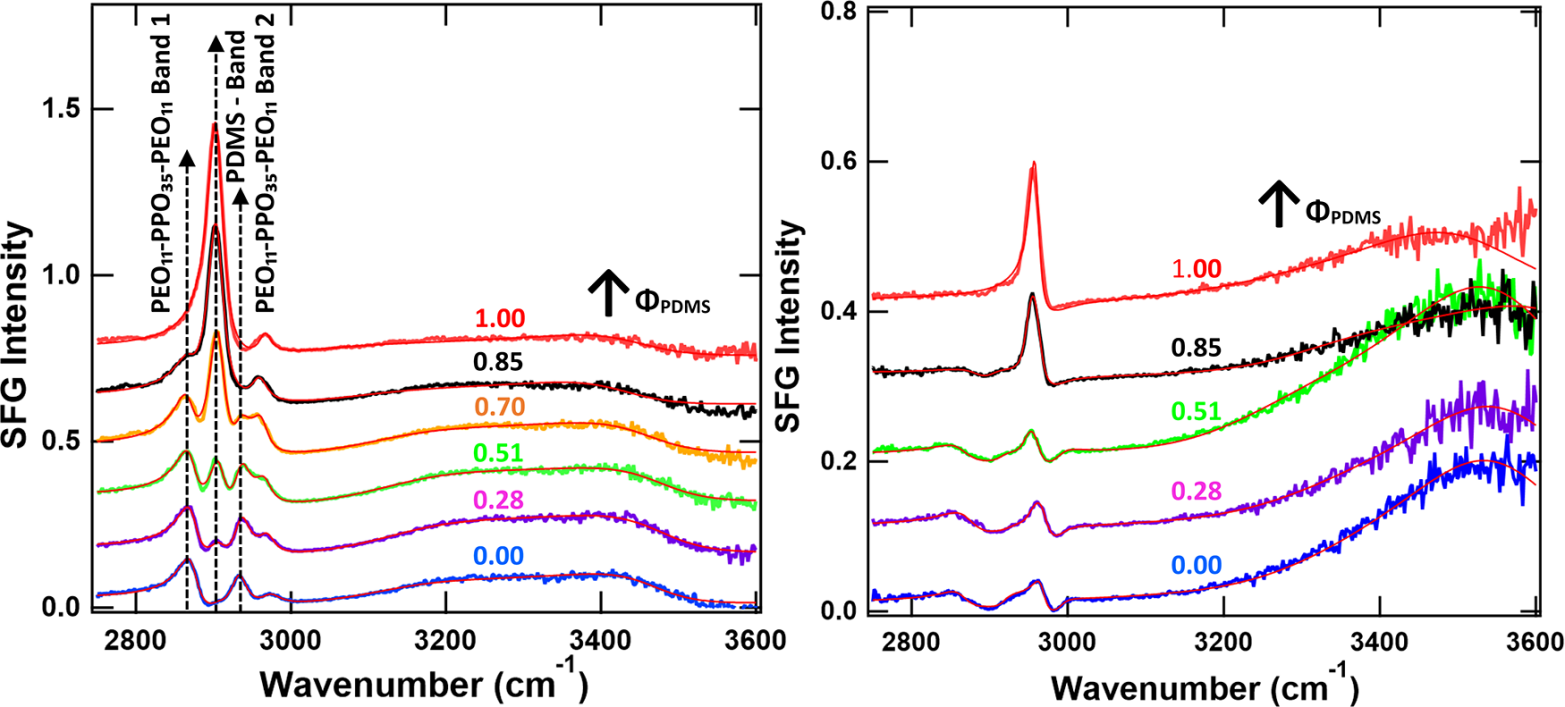
SFG spectra in SSP (A) and SPS (B) polarizations at 5 mN/m for PEO_11_–PPO_35_–PEO_11_ (blue) and PDMS (red) monolayers, as well as their blends with Φ_PDMS_ = 0.28 (magenta), 0.51 (green), 0.70 (orange) and 0.85 (black), recorded in the C–H/O-H vibrational region (2750–3600 cm^–1^). Red lines represent fits using a Lorentzian line shape model. Spectra are vertically offset for clarity. The 3 monitored bands are indicated; their assignment is provided in Table 3.

For PDMS, one characteristic band was analyzed: CH_3_ symmetric stretch in SSP, at 2904 cm^–1^ in the pure PDMS film (PDMS-band).

For PEO_11_–PPO_35_–PEO_11_ contribution, the monitored bands were:

- PEO_11_–PPO_35_–PEO_11_-band 1: CH_2_ symmetric stretch (PEO and PPO blocks) in SSP at 2873 cm^–1^ in the pure PEO_11_–PPO_35_–PEO_11_ film
- PEO_11_–PPO_35_–PEO_11_-band 2: CH_3_ symmetric stretch in SSP at 2934 cm^–1^.

### 3.3 Results

#### 3.3.1 Characterization of the First-Order Phase Transition for PEO_11_–PPO_35_–PEO_11_-Rich Blends

Two PEO_11_–PPO_35_–PEO_11_-rich mixed films, with Φ_PDMS_ = 0.28 and 0.51, were studied using NR and SFG at various surface pressures – both below and above the first-order phase transition indicated by the gray hatched region in the phase diagram, following the magenta and green arrows, respectively (Figure 2B). These measurements were performed either with dPEO_10_-dPPO_34_-dPEO_10_/PDMS or PEO_11_–PPO_35_–PEO_11_/dPDMS blends.

##### 3.3.1.1 Vertical Structure

The NR curve measured for PEO_11_–PPO_35_–PEO_11_/dPDMS mixed film with Φ_PDMS_ = 0.28, spread on an H_2_O/D_2_O subphase contrast-matched to the film, was compared to the subphase reflectivity alone at 5 mN/m (Figure 5A) and 23 mN/m (Figure 5B). Similar results at 5 and 17 mN/m for dPEO_10_–dPPO_34_–dPEO_10_/PDMS mixed film with Φ_PDMS_ = 0.51 are presented in Figure SI-11A,B. At low surface pressure (5 mN/m), the reflectivity curves for both blends closely match the subphase reflectivity. This suggests vertical homogeneity. However, above the phase transition, the NR curves deviate from the subphase reflectivity for both compositions, revealing the emergence of vertical inhomogeneity in the film structure. Combined with BAM images that show laterally homogeneous films, these results imply that the films are homogeneous both laterally and vertically below the phase transition, but become vertically inhomogeneous once the phase transition is crossed.

**Figure 5:**
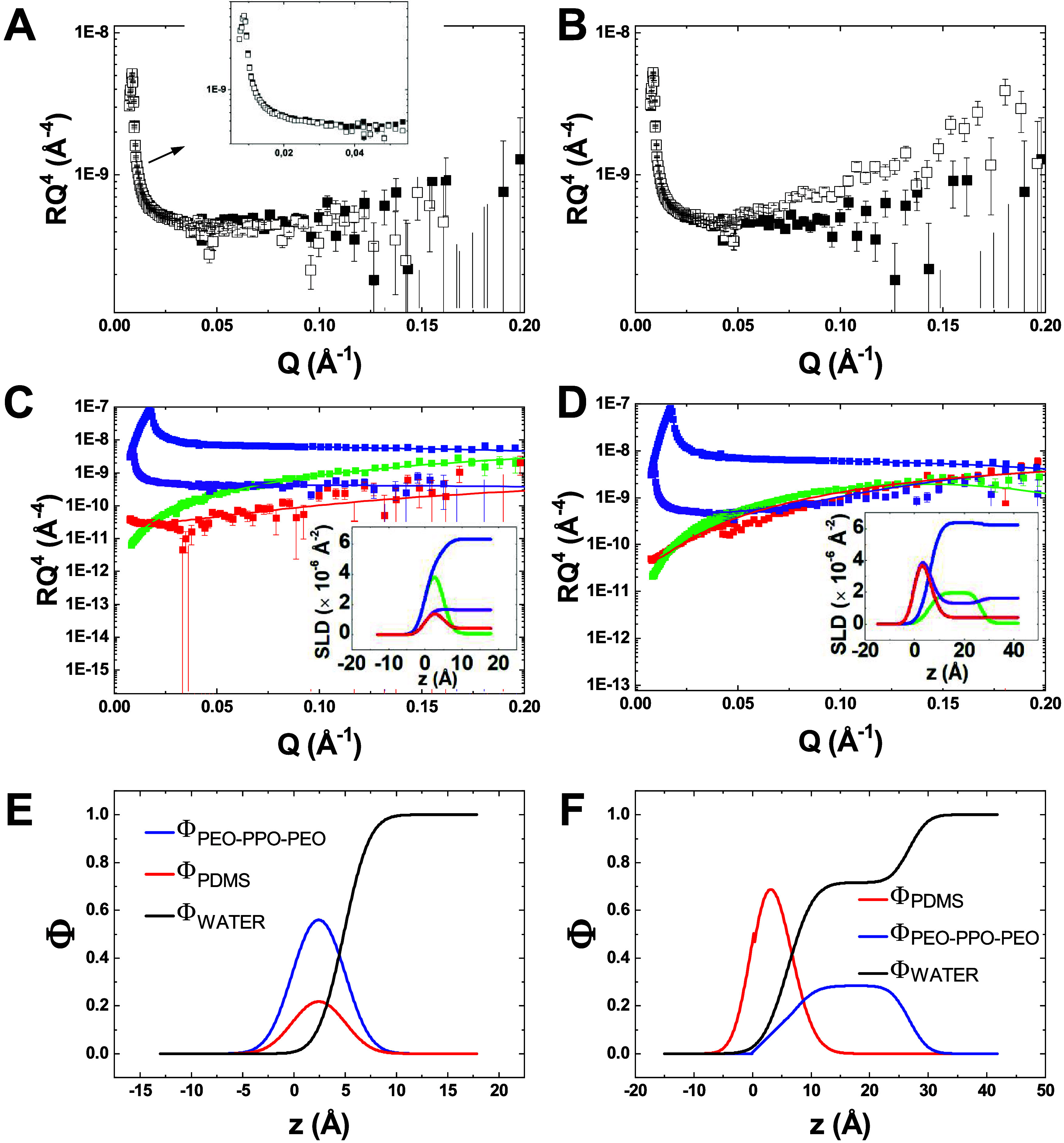
NR data for a PEO_11_–PPO_35_–PEO_11_/PDMS film with Φ_PDMS_ = 0.28 at **(**A, C, E) 5 mN/m and (B, D, F) 23 mN/m (following the magenta arrow in the phase diagram of Figure 2B). (A and B) NR curves in RQ^4^ representation as a function of wave vector transfer Q (Å^–1^) (open symbols) for PEO_11_–PPO_35_–PEO_11_/dPDMS film on a H_2_O/D_2_O subphase contrast-matched to the film if homogeneous (SLD = 1.69.10^–6^ Å^–2^) compared to the reflectivity of the subphase alone (solid symbols). (C and D) Fits of the RQ^4^ = f­(Q) NR curves obtained for dPEO_10_–dPPO_34_–dPEO_10_/PDMS film on two different H_2_O/D_2_O subphases: contrast-matched to PDMS (red), and to dPEO_10_–dPPO_34_–dPEO_10_ (blue), and for PEO_11_–PPO_35_–PEO_11_/dPDMS film on two subphases: matched to the mixed film if homogeneous (purple), and to PEO_11_–PPO_35_–PEO_11_ (green). Insets show the corresponding SLD profiles as a function of depth *z*. (E and F) Volume fraction profiles of PEO_11_–PPO_35_–PEO_11_ (blue), PDMS (red), and water (black), denoted as Φ_PEO_11_–PPO_35_–PEO_11__, Φ_PDMS_ and Φ_water_, respectively, as a function of depth.

To further investigate the structural organization of the films, the NR curves obtained for PEO_11_–PPO_35_–PEO_11_/PDMS films on four different H_2_O/D_2_O subphases were simultaneously fitted (Figure 5C,D). These systems were chosen to ensure that there is always a large contrast between the nonmatched species and both the air and the subphase. The fitting approach was based on the findings from the previous contrast-matching characterization: a single-layer model was used for homogeneous films, and a two-layer model served as a starting point for vertically inhomogeneous films, and revealed to be always sufficient to properly fit the data.

At Surface Pressure below the Phase Transition (5 mN/m): Figures 5C and SI-11C show the NR curves measured at 5 mN/m for dPEO_10_–dPPO_34_–dPEO_10_/PDMS films with Φ_PDMS_ = 0.28 and Φ_PDMS_ = 0.51, respectively. At this low surface pressure, both films exhibit vertical homogeneity, confirmed by the good agreement between the experimental curves and the single-layer model fits. The resulting SLD-depth profiles are shown in the insets of Figures 5C and SI-11C for Φ_PDMS_ = 0.28 and Φ_PDMS_ = 0.51, respectively. The extracted thicknesses of the polymer blend layers are 4.9 Å for Φ_PDMS_ = 0.28 and 7.8 Å for Φ_PDMS_ = 0.51. Both films are weakly hydrated (<20%). From these SLD-depth profiles, volume fraction depth profiles of PEO_11_–PPO_35_–PEO_11_ (Φ_PEO_11_–PPO_35_–PEO_11__), PDMS (Φ_PDMS_) and water (Φ_water_) were extracted and are presented in Figure 5E for Φ_PDMS_ = 0.28 and in Figure SI-11E for Φ_PDMS_ = 0.51. Based on the PDMS volume fraction (Φ_PDMS_ = 0.28 or 0.51), these profiles were calculated assuming that in the lower part of the film, all three components (PEO_11_–PPO_35_–PEO_11_, PDMS, and water) are present, while in the upper region, PEO_11_–PPO_35_–PEO_11_, PDMS, and air are present.^29^ The relative deviations on the polymer masses, Δ*m* (≤5%, Table SI-1), support the validity of the proposed structural model.

At Surface Pressure above the Phase Transition: the NR curves measured at 23 mN/m for the Φ_PDMS_ = 0.28 film and at 17 mN/m for the Φ_PDMS_ = 0.51 film are shown in Figure 5D and SI-11D, respectively. Starting with the Φ_PDMS_ = 0.28 film, the previously observed vertical inhomogeneity prompted the use of a two-layer model for fitting the curves. This model provided a good fit, as shown in Figure 5D, and describes a bilayer structure composed of a 6.0 Å-thick top layer of pure PDMS, and a 20.7 Å-thick bottom layer of pure PEO_11_–PPO_35_–PEO_11_, as reflected in the SLD-depth profile shown in the inset of Figure 5D. As expected from the chemical nature of the polymers, the PEO_11_–PPO_35_–PEO_11_ layer in contact with water is hydrated (72% water), while the PDMS layer is not. Several tests were performed to validate this bilayer configuration. For instance, to determine whether PDMS could instead be located at the bottom of the film, an alternative two-layer model was tested (Figure SI-12), where a pure PDMS layer was placed beneath the PEO_11_–PPO_35_–PEO_11_. This model could not reproduce the NR curves, effectively ruling out the presence of PDMS below PEO_11_–PPO_35_–PEO_11_ layer.

To conclude on the blend structure at 23 mN/m, the volume fraction depth profiles of PEO_11_–PPO_35_–PEO_11_ (Φ_PEO_11_–PPO_35_–PEO_11__), PDMS (Φ_PDMS_) and water (Φ_water_) were calculated from the SLD profiles in the inset of Figure 5D following the method developed in a previous paper, and are shown in Figure 5F.^29^ Briefly, in the bottom part of the film, where SLD varies depending on the H_2_O/D_2_O subphase, the three components (PEO_11_–PPO_35_–PEO_11_, PDMS, and water) are assumed to be present, with no air. In the upper region of the film, only PDMS and air are assumed to be present. These volume fraction profiles show that as expected, water is confined to the amphiphilic PEO_11_–PPO_35_–PEO_11_ layer, while the hydrophobic PDMS layer remains dry.

A similar bilayer structure was observed for the Φ_PDMS_ = 0.51 film at 17 mN/m above the phase transition (Figure SI-11). Due to the lower content of PEO_11_–PPO_35_–PEO_11_ in this blend, the bottom PEO_11_–PPO_35_–PEO_11_ layer is slightly thinner (16.2 Å) with same hydration (72% of water), whereas the upper PDMS layer is slightly thicker (6.7 Å) and remains dry.

For both studied compositions, the relative deviation Δ*m* (Table SI-1) on the polymer mass is higher than that obtained at lower surface pressure. This effect is particularly pronounced for the PEO_11_–PPO_35_–PEO_11_ bottom layer, where Δ*m* reaches large negative values (−73% for Φ_PDMS_ = 0.28 and −42% for Φ_PDMS_ = 0.51), while significantly smaller relative deviations are obtained for PDMS (−27% for Φ_PDMS_ = 0.28 and +2% for Φ_PDMS_ = 0.51). To better understand these discrepancies in the PEO_11_–PPO_35_–PEO_11_ bottom layer, we calculated the effective surface pressure of this layer. Knowing the mass of spread PEO_11_–PPO_35_–PEO_11_ and the through area, the mean molecular area was determined, and the corresponding surface pressure was extracted on the pure polymer isotherm. The resulting mean values are above (20 ± 1 mN/m for Φ_PDMS_ = 0.28) or close to (13 ± 3 mN/m for Φ_PDMS_ = 0.51) the collapse pressure of the pure PEO_11_–PPO_35_–PEO_11_ film (16 mN/m). Even if the polymer arrangement could be different, this observation is consistent with the onset of collapse of the PEO_11_–PPO_35_–PEO_11_ bottom layer, leading to a partial loss of material and a resulting negative increased Δ*m* (mainly for Φ_PDMS_ = 0.28). However, this phenomenon appears to be limited, as no aggregates are visible in the BAM images at the investigated surface pressures. It may also account for the slightly higher roughness at the interface between the PEO_11_–PPO_35_–PEO_11_ and PDMS layers (3.4 Å for Φ_PDMS_ = 0.28, 2.7 for Φ_PDMS_ = 0.51).

In conclusion, the first-order phase transition for PEO_11_–PPO_35_–PEO_11_-rich blends (gray hatched region in the phase diagram) corresponds to a structural transition from a homogeneous single-layer to a vertically segregated bilayer. This bilayer is characterized by a pure hydrophobic PDMS layer at the air interface and a hydrated amphiphilic PEO_11_–PPO_35_–PEO_11_ layer at the water interface.

##### 3.3.1.2 Molecular-Scale Order

To investigate the behavior of each polymer within the PEO_11_–PPO_35_–PEO_11_-rich blends with Φ_PDMS_ = 0.28 and 0.51, the evolution of the amplitude and IR frequency of three vibrational bands—originating from either PDMS or PEO_11_–PPO_35_–PEO_11_ (as listed in Table 3)—was plotted as a function of surface pressure up to film collapse, as shown in Figure 6. The evolutions of these parameters are compared to those obtained for the respective pure polymer films. For each blend, the surface pressure range corresponding to the first-order phase transition (gray hatched region in the phase diagram of Figure 2B) is indicated as a colored hatched area in Figure 6.

**Figure 6:**
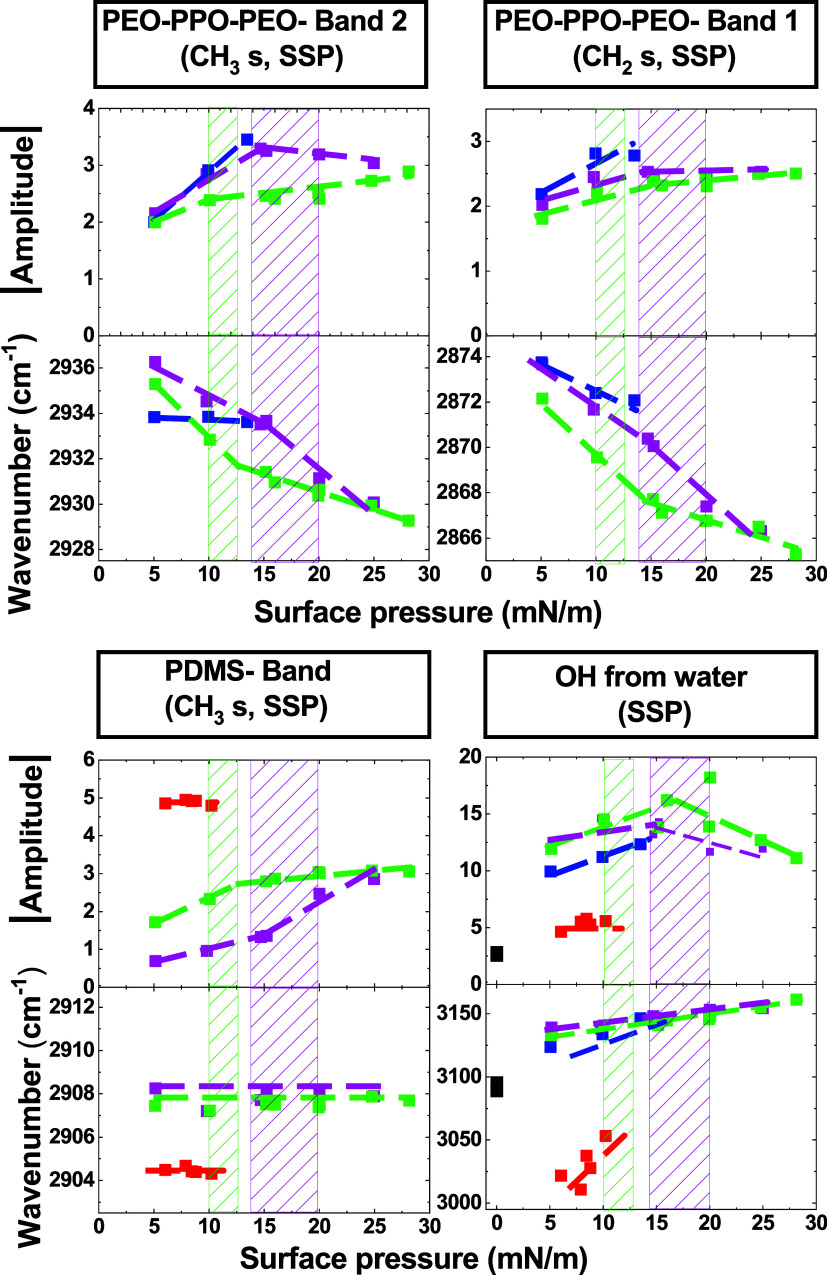
Evolution of the absolute value of the amplitude (up) and the wavenumber (IR frequency) (bottom) of characteristic PDMS (CH_3 s_), PEO_11_–PPO_35_–PEO_11_ (band1: CH_2 s_, band 2: CH_3s_) and water bands as a function of surface pressure, measured by SFG in SSP polarization (Table 3), for PEO_11_–PPO_35_–PEO_11_/PDMS blends with Φ_PDMS_ = 0.28 (dark purple) and 0.51 (light purple), compared to the same bands in pure films (PEO_11_–PPO_35_–PEO_11_ in blue, PDMS in red). The dotted lines are guides to the eye. The colored hatched areas indicate the surface pressure range corresponding to the phase transition in the phase diagram of Figure 2B (gray hatched region). Dotted lines are provided as visual guides. The black dots at zero surface pressure on the graphs associated with the OH band correspond to pure water used as a reference.

For all three bands, a noticeable change in slope is observed in the amplitude curves near the surface pressure at which the phase transition occurs in both blends. This behavior suggests a significant reorganization of both polymers within the mixed films during the transition—consistent with the transition from a homogeneous single layer to a bilayer, as demonstrated by NR data.

To account for the different amounts of each polymer present at the interface depending on the polymer volume fraction Φ_PDMS_, the amplitudes were normalized as described in the experimental section (Section 2.5). Following this normalization, any amplitude change in the blends compared with the pure polymers can be attributed to changes in the orientation of chemical bonds. The evolution of the normalized amplitudes for the three vibrational bands is shown in Figure SI-13, limited to the surface pressure range below the collapse of the pure films (16 mN/m for PEO_11_–PPO_35_–PEO_11_ and 10 mN/m for PDMS), i.e., below the phase transition. For the two PEO_11_–PPO_35_–PEO_11_-related bands (Table 3), the normalized amplitudes in both blends are all close to those of the pure PEO_11_–PPO_35_–PEO_11_ film within experimental uncertainty. This indicates that, although the blend is miscible in this range of surface pressure, the interfacial conformation of PEO_11_–PPO_35_–PEO_11_ is not significantly altered by the presence of PDMS. In contrast, for the PDMS-related band, the normalized amplitudes in the blends are markedly lower than in the pure PDMS film at low surface pressure (5 mN/m) but reach similar values upon compression to 10 mN/m. Considering the symmetric CH_3_ stretching in SSP polarization, these trends suggest that at 5 mN/m, the CH_3_ groups lie more parallel to the water surface than in the pure PDMS film. This distinct conformation of PDMS chains in the blends at low surface pressure provides clear evidence of nonideal mixing behavior in this region of the phase diagram.

Moreover, a significant shift in IR frequency is observed for the band originating from PDMS in the blends relative to the pure PDMS film, across all surface pressures (Figure 6). At low surface pressure, the combination of this frequency shift and the deviation of normalized amplitudes relative to the pure PDMS film indicates that PDMS chains in the blend adopt a different conformation, consistent with strong interactions with PEO_11_–PPO_35_–PEO_11_ and the formation of a homogeneous mixed layer as evidenced by NR. In contrast, for PEO_11_–PPO_35_–PEO_11_, below the pure film collapse pressure (16 mN/m), the IR frequencies of the three bands remain close to those measured in the pure PEO_11_–PPO_35_–PEO_11_ film. As with the normalized amplitudes, this suggests that the conformation of PEO_11_–PPO_35_–PEO_11_ is only weakly affected by PDMS, even though the polymers are miscible. Additionally, during the transition from a homogeneous single layer to a bilayer, a change in the slope of the IR frequency curves is observed for the two PEO_11_–PPO_35_–PEO_11_-related bands, whereas the PDMS-related band evolves smoothly with increasing surface pressure. This indicates that the shift from lateral to vertical PEO_11_–PPO_35_–PEO_11_–PDMS interactions primarily affects the PEO_11_–PPO_35_–PEO_11_ polymer.

Altogether, these observations confirm that both polymers exhibit distinct behaviors in the Φ_PDMS_ = 0.28 and 0.51 mixed films compared with the pure systems. Although the films appear homogeneous at surface pressures below the phase transition, the blends are not ideal. This nonideality is consistent with attractive interactions between the polymers at low surface pressure (below 9 mN/m), as also inferred from the isotherm analysis showing a negative deviation from the ideal mixing law for the mean area per repeat unit.

Finally, the O–H stretching vibration band at 3123 cm^–1^ in pure PEO_11_–PPO_35_–PEO_11_ film and 3021 cm^–1^ in pure PDMS film was monitored in SSP polarization as a function of surface pressure for both blends (Figure 6). Remarkably, in both blends, the IR frequency of the water band aligns with that of the pure PEO_11_–PPO_35_–PEO_11_ film, regardless of the surface pressure (below the collapse pressure of pure PEO_11_–PPO_35_–PEO_11_). These observations indicate that, in these PEO_11_–PPO_35_–PEO_11_-rich blends, the interaction of water occurs predominantly with PEO_11_–PPO_35_–PEO_11_. This is consistent with the amphiphilic character of PEO_11_–PPO_35_–PEO_11_ and the hydrophobic nature of PDMS, which tends to minimize interaction with water. Above the phase transition, although a direct comparison with the pure polymers is not possible due to their collapse, the IR frequency of the water band in the blends remains close to that of pure PEO_11_–PPO_35_–PEO_11_ film. This observation is consistent with the bilayer structure revealed by NR, in which the PEO_11_–PPO_35_–PEO_11_ layer remains in contact with the aqueous subphase, while PDMS is segregated toward the air interface.

#### 3.3.2 PDMS-Rich Blends: Characterization of the “Pink” Phase Transition

Two PDMS-rich mixtures, Φ_PDMS_ = 0.70 (orange arrow in Figure 2B) and Φ_PDMS_ = 0.85 (black arrow in Figure 2B), were studied below and above the phase transition represented by the pink hatched area in Figure 2B.

##### 3.3.2.1 Vertical Structure

To assess whether the films are vertically homogeneous, NR curves measured for the PEO_11_–PPO_35_–PEO_11_/dPDMS mixed films with Φ_PDMS_ = 0.70 and 0.85, spread on H_2_O/D_2_O subphase contrast-matched to the film, were compared to the subphase reflectivity at 5 mN/m and above the phase transition (20 mN/m for Φ_PDMS_ = 0.70 and 15 mN/m for Φ_PDMS_ = 0.85). Figures 7A,B and SI-14A,B show the curves measured for Φ_PDMS_ = 0.70 and Φ_PDMS_ = 0.85, respectively. For both compositions, and at all surface pressures investigated, a deviation—ranging from slight to significant—is observed between the NR curves and the subphase reflectivity, indicating vertical inhomogeneity in the films.

**Figure 7:**
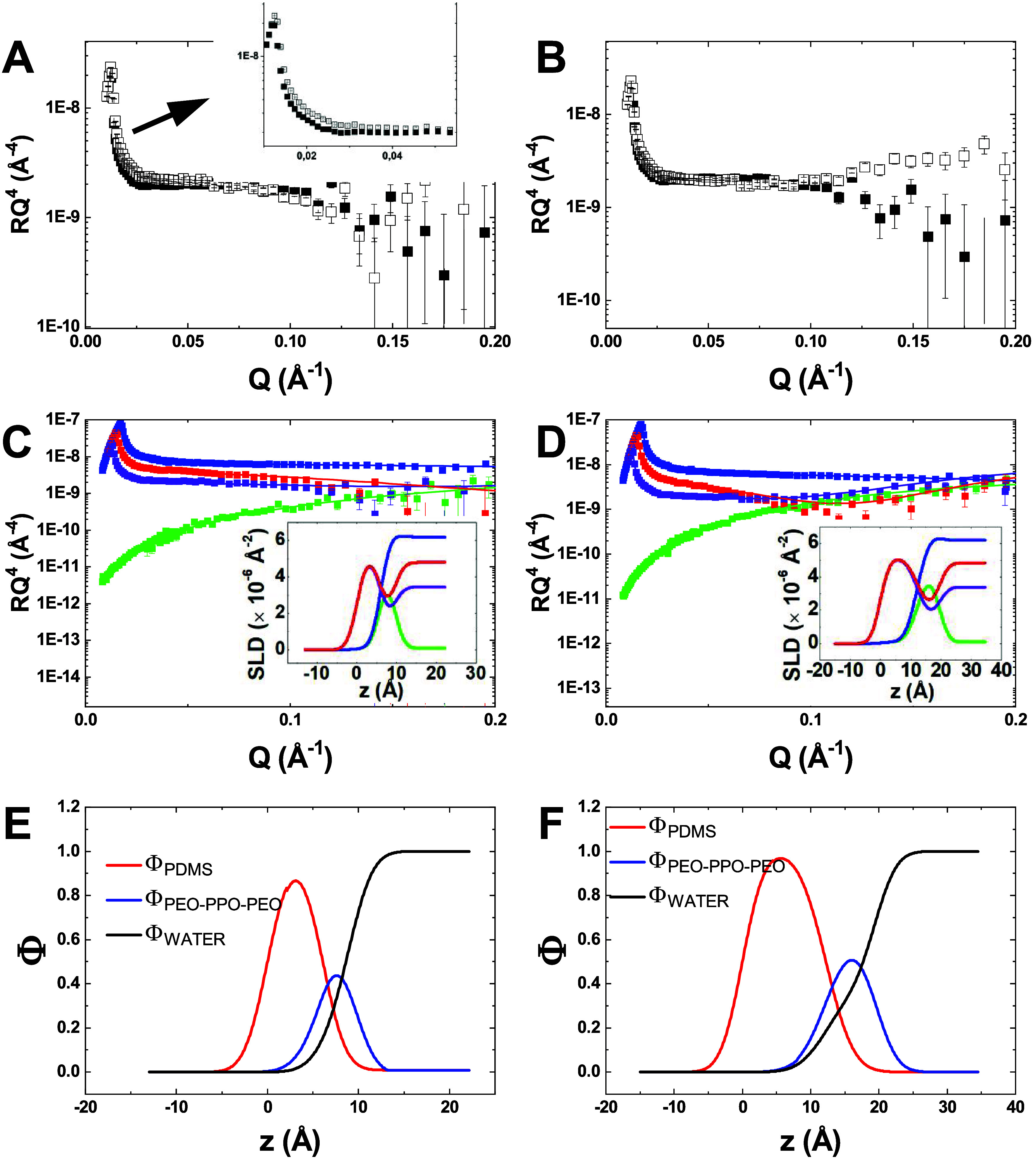
NR data for a PEO_11_–PPO_35_–PEO_11_/PDMS film with Φ_PDMS_ = 0.70 at (A, C, E) 5 mN/m and (B, D, F) 20 mN/m (following the orange arrow in the phase diagram of Figure 2B). (A and B) NR curves in RQ^4^ representation as a function of wave vector transfer Q (Å^–1^) (open symbols) for a PEO_11_–PPO_35_–PEO_11_/dPDMS film on a H_2_O/D_2_O subphase contrast-matched to the film if homogeneous (SLD = 3.67.10^–6^ Å^–2^) compared to reflectivity of the subphase alone (solid symbols). (C and D) Fits of the RQ^4^ = f­(Q) NR curves obtained for dPEO_10_–dPPO_34_–dPEO_10_/PDMS film on two different H_2_O/D_2_O subphases: contrast-matched to PDMS (green), and to dPEO_10_–dPPO_34_–dPEO_10_ (blue), and for PEO_11_–PPO_35_–PEO_11_/dPDMS film on two subphases: matched to the mixed film if homogeneous (purple), and to dPDMS (red). Insets show the corresponding SLD profiles as a function of depth *z*. (E and F) Volume fraction profiles of PEO_11_–PPO_35_–PEO_11_ (blue), PDMS (red), and water (black), denoted as Φ_PEO_11_–PPO_35_–PEO_11__, Φ_PDMS_ and Φ_water_, respectively, as a function of depth.

To investigate this vertical inhomogeneity in more detail, Figures 7C,D show the NR curves for the Φ_PDMS_ = 0.70 PEO_11_–PPO_35_–PEO_11_/PDMS film measured at 5 and 20 mN/m, with either dPEO_10_–dPPO_34_–dPEO_10_ or dPDMS in the blends, respectively, using four different H_2_O/D_2_O subphases: one contrast-matched to PDMS, one to dPEO_10_–dPPO_34_–dPEO_10_, one to dPDMS, and one to the mixed film if homogeneous. As vertical inhomogeneity was observed at both surface pressures, a two-layer model was first applied to fit the data, following the same methodology used for the vertically segregated Φ_PDMS_ = 0.28 PEO_11_–PPO_35_–PEO_11_/PDMS film at 23 mN/m. This model, consisting of a pure PDMS layer atop a pure PEO_11_–PPO_35_–PEO_11_ layer provided a good fit to the data at both surface pressures. The corresponding SLD-depth profiles, shown in the insets of Figures 7C, D, were used to calculate the volume fraction depth profiles of PEO_11_–PPO_35_–PEO_11_ (Φ_PEO_11_–PPO_35_–PEO_11__), PDMS (Φ_PDMS_), and water (Φ_water_), which are presented in Figure 7E (5 mN/m) and Figure 7F (20 mN/m). Across the phase transition, the thickness of the PEO_11_–PPO_35_–PEO_11_ bottom layer varies from 3.1 Å at 5 mN/m to 7.3 Å at 20 mN/m, with a moderate hydration level. The upper PDMS layer exhibits a notable change, with its thickness doubling, from 6.1 Å at 5 mN/m to 12.2 Å at 20 mN/m. As expected for hydrophobic PDMS, this layer remains nonhydrated.

For the Φ_PDMS_ = 0.85 film, NR measurements were performed using dPDMS only, on two subphases: one contrast-matched to dPDMS, and another matched to the mixed film if homogeneous (Figure SI-14C,D). At both surface pressures, the NR data were best fitted using a bilayer model consisting of a pure PDMS layer on top of a pure PEO_11_–PPO_35_–PEO_11_ layer. The volume fractions of polymers and water derived from the fits are presented in Figure SI-14E,F for surface pressures of 5 and 15 mN/m, respectively. At both surface pressures, the two layers appear to be nonhydrated. The thickness of the upper PDMS layer increases from 9.8 Å at 5 mN/m to 13.9 Å at 15 mN/m. Meanwhile, the PEO_11_–PPO_35_–PEO_11_ bottom layer remains extremely thin, consistent with the low proportion of amphiphilic polymer in the blend. At 5 mN/m, to obtain reliable results, its thickness was calculated based on the volume fraction of PDMS introduced in the film (Φ_PDMS_ = 0.85). The value of 1.7 Å is of the same order as the roughness at the film–air interface (2.0 Å). It is worth noting that the NR curves at 5 mN/m can also be reasonably fitted using a single-layer model with a SLD close to that of pure PDMS, but with a pronounced roughness of 5 Å at the film-water interface. This supports the presence of PEO_11_–PPO_35_–PEO_11_ in contact with water, embedded within the interfacial roughness of the PDMS layer. Above the phase transition, at 20 mN/m, the PEO_11_–PPO_35_–PEO_11_ bottom layer becomes slightly thicker, reaching 3.1 Å.

For both studied compositions, the relative deviation Δ*m* (Table SI-1) on the polymer mass shows very low values at 5 mN/m (≤14%). Above the phase transition, the values are generally slightly higher but remain of the same order of magnitude as those obtained for the pure films at high surface pressure, except for PDMS at 15 mN/m for Φ_PDMS_ = 0.85, where a large negative relative deviation is observed (−47%). This discrepancy could be attributed to bilayer compression effects leading to an effective surface pressure of the PDMS top layer of 9.4 ± 0.5 mN/m, which is close to the collapse of the pure PDMS film (10 mN/m). Although no aggregates were detected by BAM, the onset of their formation could explain the lower PDMS content observed in the bilayer.

For PDMS-rich blends, the results clearly show that the films maintain a bilayer structure at all measured surface pressures, consisting of a pure PDMS upper layer and a pure PEO_11_–PPO_35_–PEO_11_ bottom layer. No significant structural reorganization is observed across the phase transition (pink hatched region in the phase diagram). Rather, the transition is primarily characterized by a thickening of the PDMS upper layer—similar to what is observed for the pure PDMS film.

##### 3.3.2.2 Molecular-Scale Order

Figure 8 shows the evolution, as a function of surface pressure up to film collapse, of the amplitude and IR frequency of three vibrational bands associated exclusively with either PDMS or PEO_11_–PPO_35_–PEO_11_ (Table 3), for the Φ_PDMS_ = 0.70 and 0.85 PDMS-rich blends, alongside the corresponding data for the pure polymer films. For all bands, the amplitude evolves continuously with surface pressure, without any noticeable change in slope at the first-order phase transition (indicated by the colored hatched areas). The IR frequencies of all bands are slightly shifted compared to those observed in the pure films, suggesting a different chemical environment for each polymer within the blends. Such behavior is consistent with the bilayer structure identified in these PDMS-rich blends at all surface pressures. The contact between PEO_11_–PPO_35_–PEO_11_ (in the bottom layer) and PDMS (in the top layer) necessarily leads to polymer interactions. The absence of any significant change in amplitude slope across the phase transition may be explained by the fact that this transition primarily involves a thickening of the PDMS top layer, without major structural reorganization. This is further supported by the constant amplitude of PDMS-related bands in the pure PDMS film across its phase transition.

**Figure 8:**
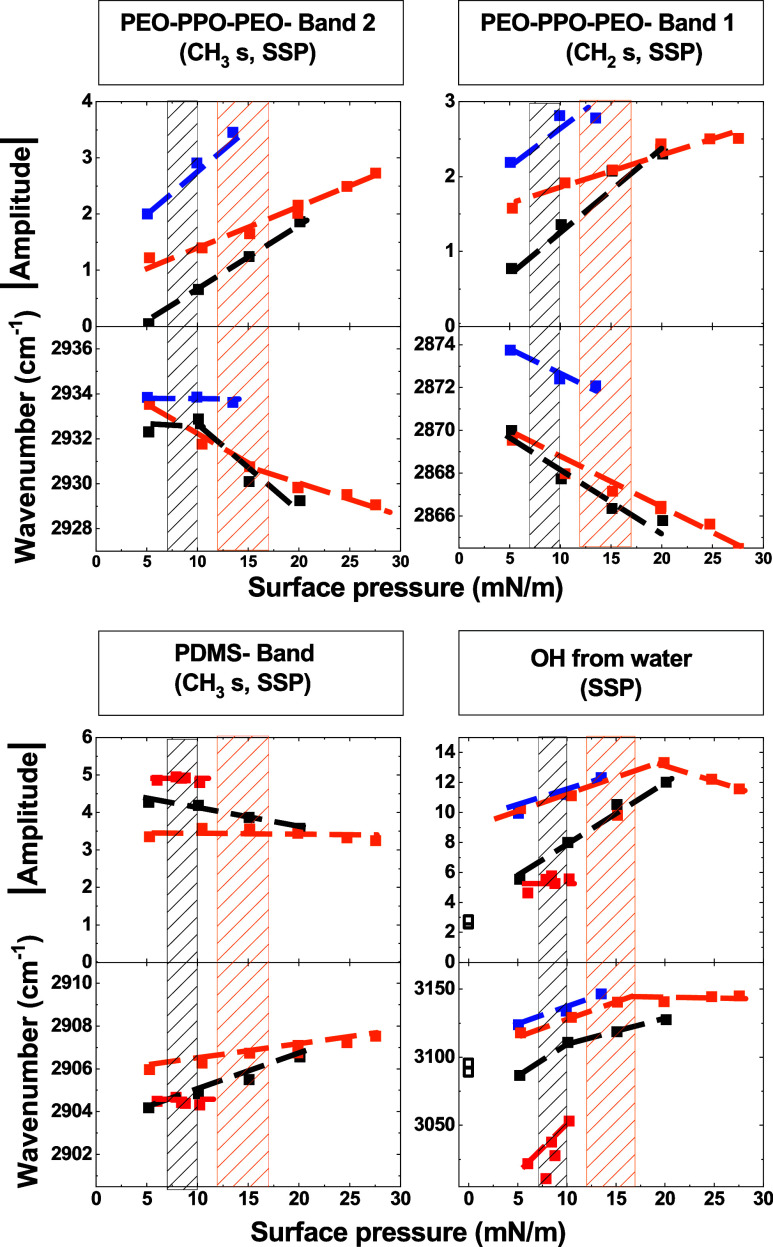
Evolution of the absolute value of the amplitude (up) and the wavenumber (IR frequency) (bottom) of characteristic PDMS, PEO_11_–PPO_35_–PEO_11_ and water bands as a function of surface pressure, measured by SFG in SSP polarization (Table 3), for PEO_11_–PPO_35_–PEO_11_/PDMS blends with Φ_PDMS_ = 0.70 (in orange) and 0.85 (in black), compared to the same bands in pure films (PEO_11_–PPO_35_–PEO_11_ in blue, PDMS in red). The colored hatched areas indicate the surface pressure range corresponding to the phase transition shown in the phase diagram of Figure 2B (pink hatched region). Dotted lines are provided as visual guides. The hollow black dots at zero surface pressure on the graphs associated with the OH band correspond to pure water used as a reference.

Figure SI-15 presents the evolution of the normalized amplitudes of three vibrational bands (from both polymers) as a function of surface pressure, up to the collapse point of the pure films. The normalization procedure accounts for the different amounts of each polymer present in the blends and in the pure films, allowing the analysis to focus specifically on molecular orientation effects. For PEO_11_–PPO_35_–PEO_11_, the normalized amplitude of the symmetric CH_2_ vibration arising from both PEO and PPO blocks (Band 1) is similar in the blends and the pure film. This indicates that the average orientation of CH_2_ groups remains largely unchanged. In contrast, the normalized amplitude of the band associated with the symmetric stretch (Band 2) of the CH_3_ groups in the PPO blocks is lower than in pure PEO_11_–PPO_35_–PEO_11_. This suggests that the CH_3_ groups are oriented more parallel to the interface than in pure PEO_11_–PPO_35_–PEO_11_ film. For PDMS below the collapse pressure of the pure film, the normalized amplitudes of the symmetric CH_3_ vibration (SSP polarization) in the blends are significantly lower than those in the pure film. This observation indicates that, at low surface pressure, the CH_3_ groups lie more parallel to the water surface than in the pure PDMS film.

Finally, Figure 8 presents the surface-pressure-dependent evolution of the amplitude and IR frequency of the strong O–H stretching vibration. Distinct behaviors are observed for the two blends. In the Φ_PDMS_ = 0.70 blend, both amplitude and frequency closely match those of the pure PEO_11_–PPO_35_–PEO_11_ film across the entire pressure range, indicating predominant interaction of water with PEO_11_–PPO_35_–PEO_11_. This is consistent with the bilayer structure, where a pure PEO_11_–PPO_35_–PEO_11_ layer remains in contact with water throughout compression. In contrast, for the Φ_PDMS_ = 0.85 blend, the amplitude and IR frequency of the O–H vibration fall between the values observed for the two pure films at 5 mN/m but shift closer to those of pure PEO_11_–PPO_35_–PEO_11_ above the phase transition. This behavior can be attributed to the low proportion of PEO_11_–PPO_35_–PEO_11_ in the blend, resulting in a very thin PEO_11_–PPO_35_–PEO_11_ bottom layer, as revealed by NR. This effect is particularly pronounced at low surface pressure, where the PEO_11_–PPO_35_–PEO_11_ is embedded within the interfacial roughness of the PDMS top layer.

### 4 Discussion

The combination of NR and SFG provides a coherent picture of the miscibility behavior and structure of PEO_11_–PPO_35_–PEO_11_/PDMS blends at the air–water interface as a function of composition and surface pressure. For PEO_11_–PPO_35_–PEO_11_-rich blends, NR demonstrates a transition from a homogeneous monolayer to a vertically segregated bilayer as the surface pressure increases. The bilayer consists of a hydrophobic PDMS layer on top of a hydrated PEO_11_–PPO_35_–PEO_11_ layer in contact with water. A similar structural evolution—from homogeneous monolayer to bilayer—is also observed at low surface pressure when the PDMS content is increased from Φ_PDMS_ = 0.51 to 0.70, as determined by NR. This indicates that, in the phase diagram shown in Figure 2B, the transition for PEO_11_–PPO_35_–PEO_11_-rich blends extends down to zero surface pressure at around Φ_PDMS_ ∼ 0.6. This is depicted in the Figure 2B as a black dotted line, which is only a guide to the eye. This could not be easily identified from compressibility measurements performed at discrete compositions. In PDMS-rich blends, a bilayer structure is present over the entire surface pressure range, with the first-order phase transition primarily associated with a thickening of the PDMS layer. This transition is similar in nature to that of pure PDMS, but its onset is influenced by interactions with the underlying PEO_11_–PPO_35_–PEO_11_ layer in contact with water. Overall, the phase diagram derived from compressibility measurements can be divided into three regions, and the corresponding structures are summarized in Figure 2C. It is also worth noting that isotherm measurements during compression–expansion cycles show that both phase transitions are reversible, indicating that even the monolayer-to-bilayer transition can be reversed upon decompression.

In addition, SFG spectroscopy provides molecular-scale evidence supporting this picture and offers a more comprehensive understanding of polymer–polymer and polymer–water interactions. At low surface pressures, below the onset of the two phase transitions (i.e., below the collapse pressures of the two pure films), the conformation of PEO_11_–PPO_35_–PEO_11_ chains in the blends is affected differently depending on whether the blend is miscible (homogeneous monolayer, region 1) or vertically segregated (bilayer, region 2). In region 1, the PEO_11_–PPO_35_–PEO_11_ chains largely retain the same conformation as in pure film and are not significantly influenced by lateral interaction with PDMS, likely due to their strong interaction with water as revealed by SFG. In contrast, in region 2, the conformation of the PEO_11_–PPO_35_–PEO_11_ chains in the bottom layer differs from that in the pure film, indicating that the interaction with the PDMS top layer becomes stronger than the PEO_11_–PPO_35_–PEO_11_-water interaction. Conversely, PDMS chains display a different arrangement in the blends compared to the pure film, with CH_3_ groups lying more parallel to the surface, regardless of whether the structure is a monolayer or a bilayer. This demonstrates that PDMS is more strongly affected by the presence of the PEO_11_–PPO_35_–PEO_11_, either laterally in agreement with its poor affinity with water, or beneath it.

Overall, these results emphasize that, despite their strong difference in hydrophobicity, PEO_11_–PPO_35_–PEO_11_ and PDMS exhibit miscibility over a range of compositions and surface pressures (Φ_PDMS_ < 0.6 and π < 10–15 mN/m depending on composition). Importantly, the blend is not ideal, with attractive interactions between the two polymers, as revealed by both the additivity law and SFG analysis. Upon increasing PDMS content or surface pressure, the blend becomes unstable; owing to the poor affinity of PDMS for water, this instability results in complete vertical segregation rather than lateral phase separation. This study also highlights the significantly different behavior of polymer blends in Langmuir films compared with bulk systems, for which a very limited number of polymer blends are miscible. To our knowledge, no study on the bulk phase diagram of PDMS/PEO–PPO–PEO blends has been reported, which is likely due to the strong immiscibility of these polymers, which arises from the large difference in their solubility parameters.^2^

### 5 Conclusion

In this work, we performed a multiscale analysis of PEO_11_–PPO_35_–PEO_11_/PDMS blends at the air–water interface, combining surface pressure–area isotherms, Brewster angle microscopy (BAM), neutron reflectometry (NR), and sum-frequency generation (SFG) spectroscopy. This approach allowed us to determine both the lateral and vertical structure of the films and to unravel polymer–polymer and polymer–water interactions. Through a detailed analysis of compressibility curves derived from the isotherms, we constructed a surface pressure–composition phase diagram revealing two distinct first-order phase transitions. While BAM detected no lateral phase separation at any surface pressure or composition, NR revealed that in PEO_11_–PPO_35_–PEO_11_-rich blends, the phase transition corresponds to a purely vertical segregation from an initially homogeneous blend. In contrast, PDMS-rich blends exhibited monolayer instability even at low surface pressures, leading to bilayer formation in which the PDMS top layer undergoes the same transition as observed in the pure PDMS film. This analysis led to the identification of three distinct regions in the phase diagram. Importantly, the blend can be stabilized as a monolayer over a wide range of compositions and surface pressures, due to the strong interactions between PEO_11_–PPO_35_–PEO_11_ and water. This work provides new insights into polymer miscibility in 2D configurations and opens avenues for designing functional nanostructured coatings.

## Supplementary Material



## Notes

Relative error Δ*m* on the polymer mass *m* deduced from NR data (Table SI-1), isotherms for PEO_11_–PPO_35_–PEO_11_/PDMS films during two cycles of compression–expansion cycles (Figure SI-1), BAM images for PEO_11_–PPO_35_–PEO_11_/PDMS Langmuir films (Figure SI-2), NR data for a pure dPEO_10_–dPPO_34_–dPEO_10_ film (Figure SI-3), SFG spectra in SPS polarization for PEO_11_–PPO_35_–PEO_11_ and PDMS Langmuir films (Figure SI-4), spectra of the imaginary and real parts of χ^(2)^ in SSP polarization for PEO_11_–PPO_35_–PEO_11_ and PDMS Langmuir films (Figure SI-5), spectra of the imaginary and real parts of χ^(2)^ in SPS polarization for PEO_11_–PPO_35_–PEO_11_ and PDMS Langmuir films (Figure SI-6), SSP/SPS and s/as SFG amplitude ratios of the CH_3_ or CH_2_ vibration for the PEO_11_–PPO_35_–PEO_11_ Langmuir film (Figure SI-7), NR data for pure PDMS Langmuir films (Figure SI-8), SSP/SPS and s/as SFG amplitude ratios of the CH_3_ vibration for the PDMS Langmuir film (Figure SI-9), SFG spectra in SSP and SPS polarizations at various surface pressures for PEO_11_–PPO_35_–PEO_11_/PDMS monolayers (Figure SI-10), NR data for a PEO_11_–PPO_35_–PEO_11_/PDMS film with Φ_PDMS_ = 0.51 (Figure SI-11), fits of the NR data for a PEO_11_–PPO_35_–PEO_11_/PDMS Langmuir film with Φ_PDMS_ = 0.28 (23 mN/m) using a model assuming a pure PEO_11_–PPO_35_–PEO_11_ layer on top of a pure PDMS layer (Figure SI-12), normalized amplitude of characteristic SFG bands as a function of surface pressure for PEO_11_–PPO_35_–PEO_11_/PDMS blends with Φ_PDMS_ = 0.28 and 0.51 (Figure SI-13), NR data for a PEO_11_–PPO_35_–PEO_11_/dPDMS film with Φ_PDMS_ = 0.85 (Figure SI-14), normalized amplitude of characteristic SFG bands as a function of surface pressure for PEO_11_–PPO_35_–PEO_11_/PDMS blends with Φ_PDMS_ = 0.70 and 0.85 (Figure SI-15) (PDF)
