# Supplementary material for: Phase Diagram of 2D Poly(Ethylene Oxide)-block-Poly(Propylene Oxide)-block-Poly(Ethylene Oxide)—Poly(Dimethylsiloxane) Blends: A Combined Neutron Reflectometry and Sum Frequency Generation Study
Source: Langmuir. 2026 Mar 16;42(12):8548–66. doi: 10.1021/acs.langmuir.5c06510 (PMC13045023; doi:10.1021/acs.langmuir.5c06510)
Supplement: Supplementary file 1 [file la5c06510_si_001.pdf]

Supporting Information

Phase diagram of 2D poly(ethylene oxide)-block-poly(propylene oxide)-block-poly(ethylene oxide) – polydimethylsiloxane blends: A combined neutron reflectometry and sum frequency generation study

Aurély Araminthe[†], Alae El Haitami[†], Bence Kővágó[‡], Fabrice Cousin[&], Pablo Sanchez-Puga[‖], John Robert Peter Webster[⊥], Philipp Gutfreund[‖], Ellen H. G. Backus[‡] and Sophie Cantin[†]

[†]*CY Cergy Paris Université, LPPI F95000 Cergy, France,*

[‡]*Institute of Physical Chemistry, Faculty of Chemistry, University of Vienna, Währinger Strasse 42, 1090 Vienna, Austria and University of Vienna, Vienna Doctoral School in Chemistry (DoSChem), Währinger Strasse 42, 1090 Vienna, Austria*

[&]*Laboratoire Léon Brillouin, Université Paris-Saclay, CEA-CNRS UMR 12, F-91191 Gif-sur-Yvette, France,*

[‖]*Institut Laue-Langevin, 71 Avenue des Martyrs, 38000 Grenoble, France*

[⊥] *ISIS Neutron & Muon Source, Rutherford Appleton Lab, STFC, Didcot OX11 0QX, England*

Φ_{PDMS}	X_{PDMS}	Surface pressure	Vertical structure	Δ*m*%	
				PEO_{11}-PPO_{35}-PEO_{11}	PDMS
**1**	**1**	5.0	Single layer		-6
		9.2<br>A = 14.1 Å^2	Single layer		-7
		9.2<br>A = 7.7 Å^2	Single layer		-26
**0**	**0**	5.0	Single layer	-19	
		10.0	Single layer	-27	
**0.28**	**0.2**	5.0	Single layer	+2	+5
		23.0	Bilayer: Upper layer<br>Bottom layer	-73	-27
**0.51**	**0.4**	5.0	Single layer	-4	0
		17.0	Bilayer: Upper layer<br>Bottom layer	-42	+2
**0.70**	**0.6**	5.0	Bilayer: Upper layer<br>Bottom layer	-5	+4
		20.0	Bilayer: Upper layer<br>Bottom layer	-27	-24
**0.85**	**0.8**	5.0	Bilayer: Upper layer<br>Bottom layer	+13	+14
		15.0	Bilayer: Upper layer<br>Bottom layer	-16	-47

Table SI-1. Relative error Δ*m* on the polymer mass *m* deduced from NR data, with respect to the spread mass m_S at the air-water interface: $\Delta m = (m - m_S) / m_S$, for pure PEO_{11}-PPO_{35}-PEO_{11} (Φ_{PDMS} =0) and pure PDMS (Φ_{PDMS} =1) films, as well as for the four mixed films studied, at different surface pressures. One should mention that the error on Δ*m* increases above the phase transitions (± 10%) due to the lower reproducibility of the isotherms close to the collapse surface pressure.

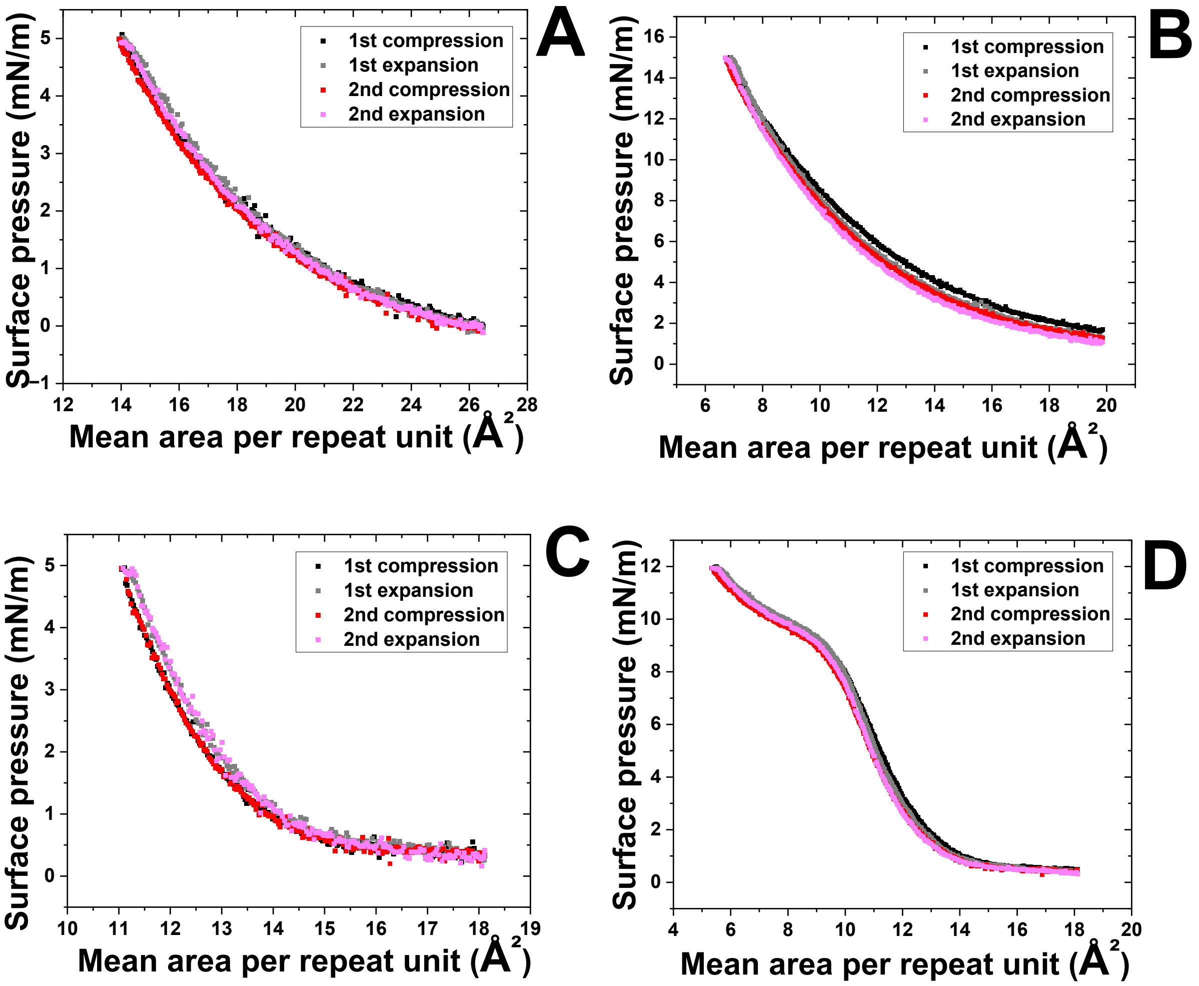

Figure SI-1. Surface pressure-mean area per repeat unit isotherms for PEO_{11}-PPO_{35}-PEO_{11}/PDMS films with Φ_{PDMS}= 0.51 (**A**, **B**) and 0.85 (**C**, **D**) during two successive cycles of compression-expansion experiments, either up to 5 mN/m (**A**, **C**) or to a surface pressure above the phase transition (15 mN/m for Φ_{PDMS}= 0.51 (**B**), 12 mN/m Φ_{PDMS}= 0.85 (**D**)).

(A) **(B)** **(C)**

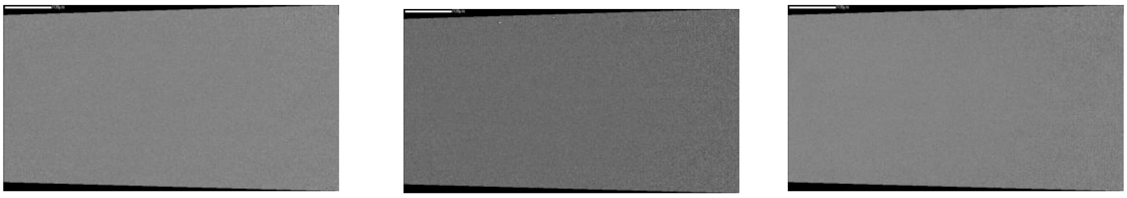

Figure SI-2. BAM images for PEO_{11}-PPO_{35}-PEO_{11}/PDMS Langmuir films in region 1 (**A**), region 2 (**B**) and region 3 (**C**) of the phase diagram shown in Figure 2B. The scale bar represents 100 µm.

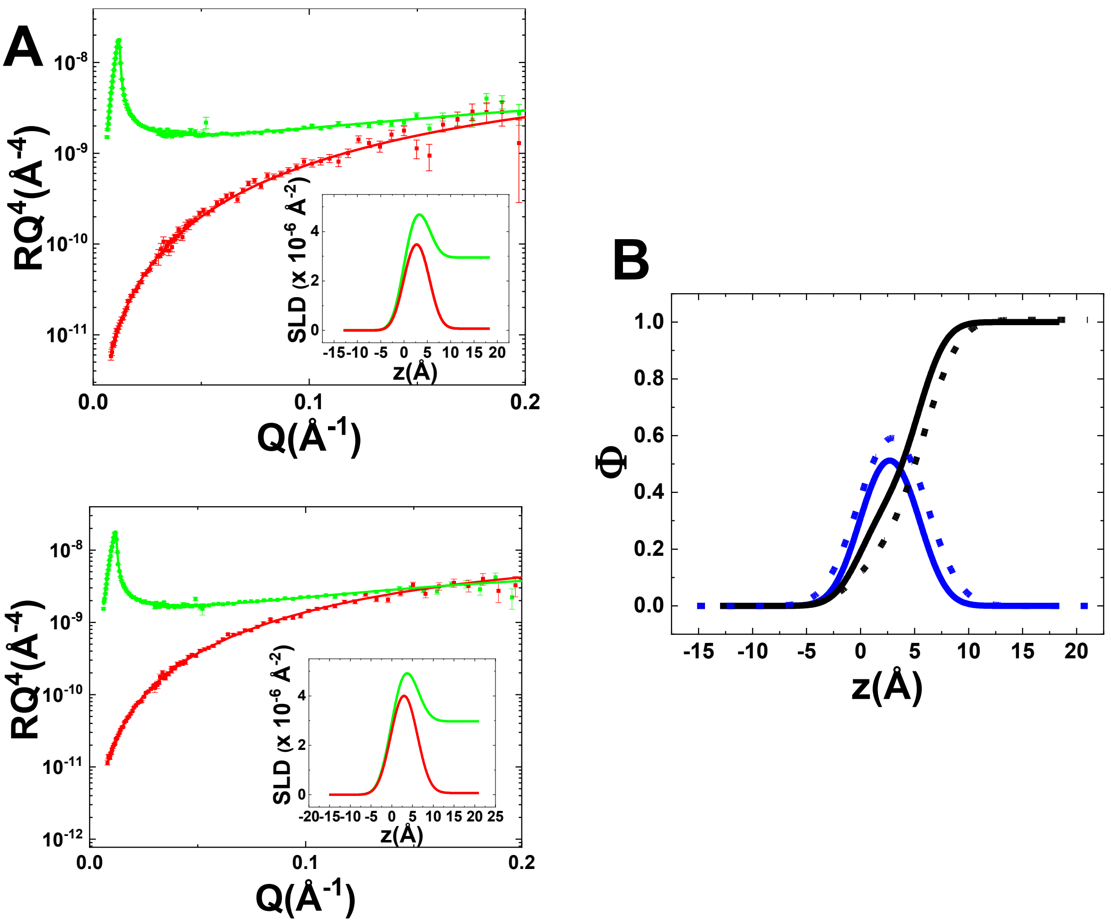

Figure SI-3. **A-** Fits of the NR curves in RQ^4 representation as a function of wave vector transfer Q ($Å^{-1}$) for a pure $dPEO_{10}$-$dPPO_{34}$-$dPEO_{10}$ film at 5 mN/m (top) and 10 mN/m (bottom), measured on two H_2O/D_2O subphases: one contrast-matched to PDMS (red), and one with an intermediate SLD of 3.10^{-6} $Å^{-2}$ (green). The insets show the corresponding SLD profile as a function of depth z. **B-** Volume fraction profiles of PEO_{11}-PPO_{35}-PEO_{11} (blue) and water (black), $\Phi_{PEO_{11}\text{-}PPO_{35}\text{-}PEO_{11}}$ and Φ_{water}, as a function of depth, at 5 mN/m (solid line) and 10 mN/m (dashed line).

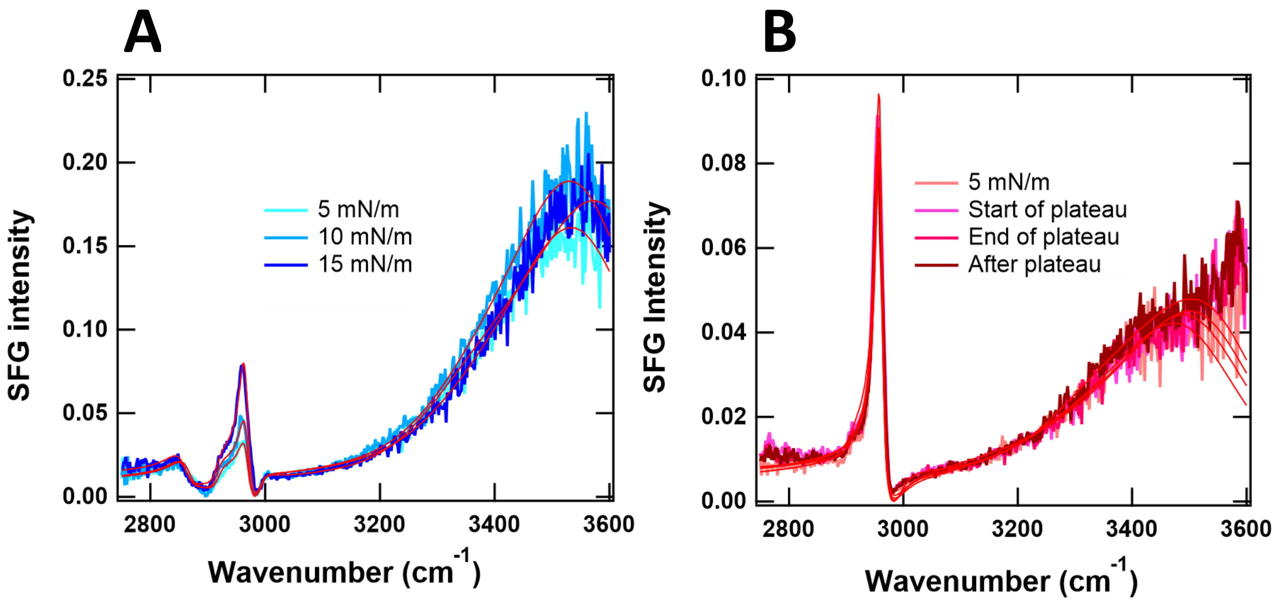

Figure SI-4. SFG spectra in SPS polarization at different surface pressures in the C-H/O-H vibrational region (2750-3600 cm^{-1}) for PEO_{11}-PPO_{35}-PEO_{11} (**A**) and PDMS (**B**) Langmuir films. The spectra are fitted using a Lorentzian lineshape model (solid lines).

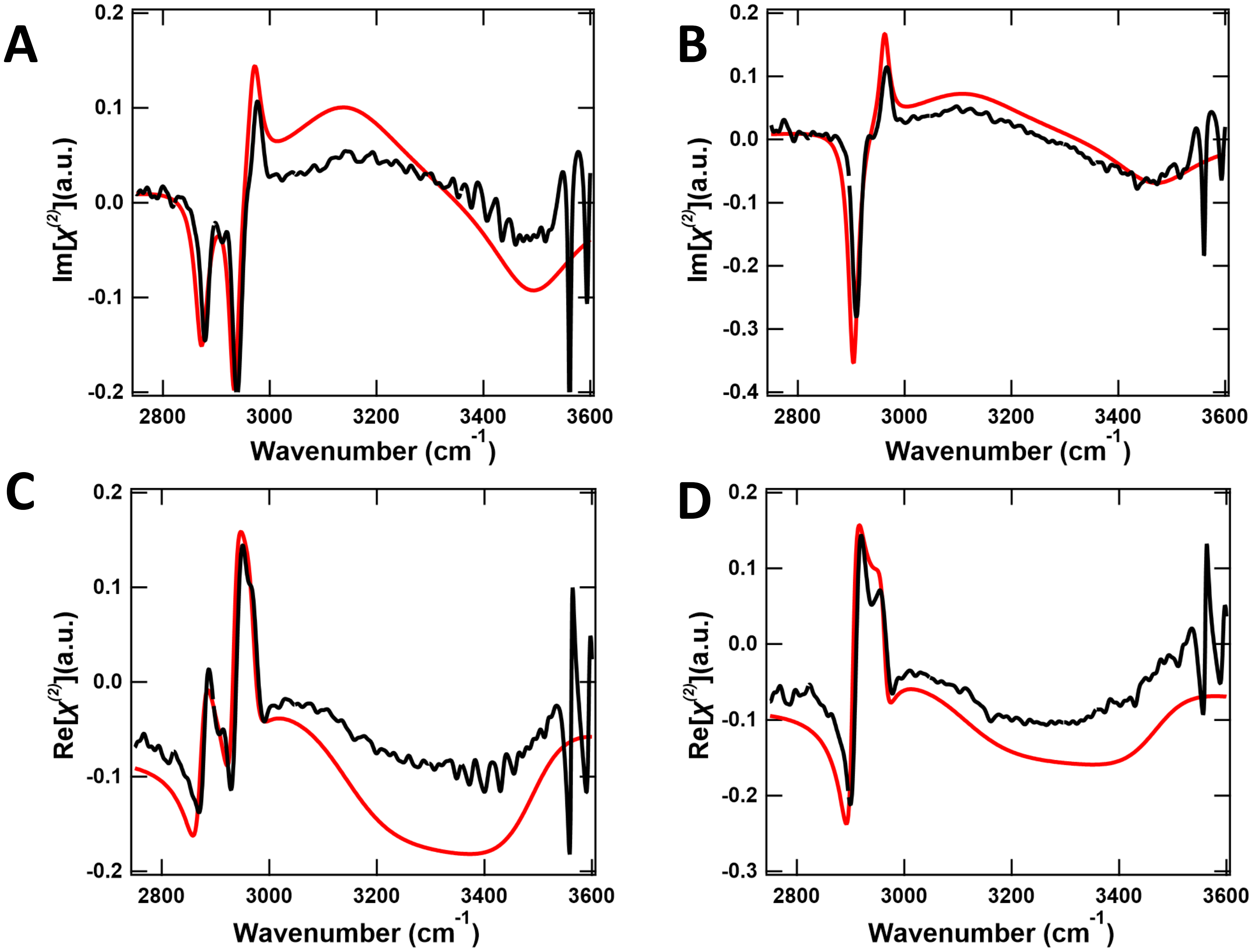

Figure SI-5. Spectra of the imaginary (**A** and **B**) and real (**C** and **D**) parts of simulated (red line) and measured (black line) non-linear susceptibility $\chi^{(2)}$ as a function of wavenumber in SSP polarization for PEO_{11}-PPO_{35}-PEO_{11} (**A** and **C**) and PDMS (**B** and **D**) Langmuir films.

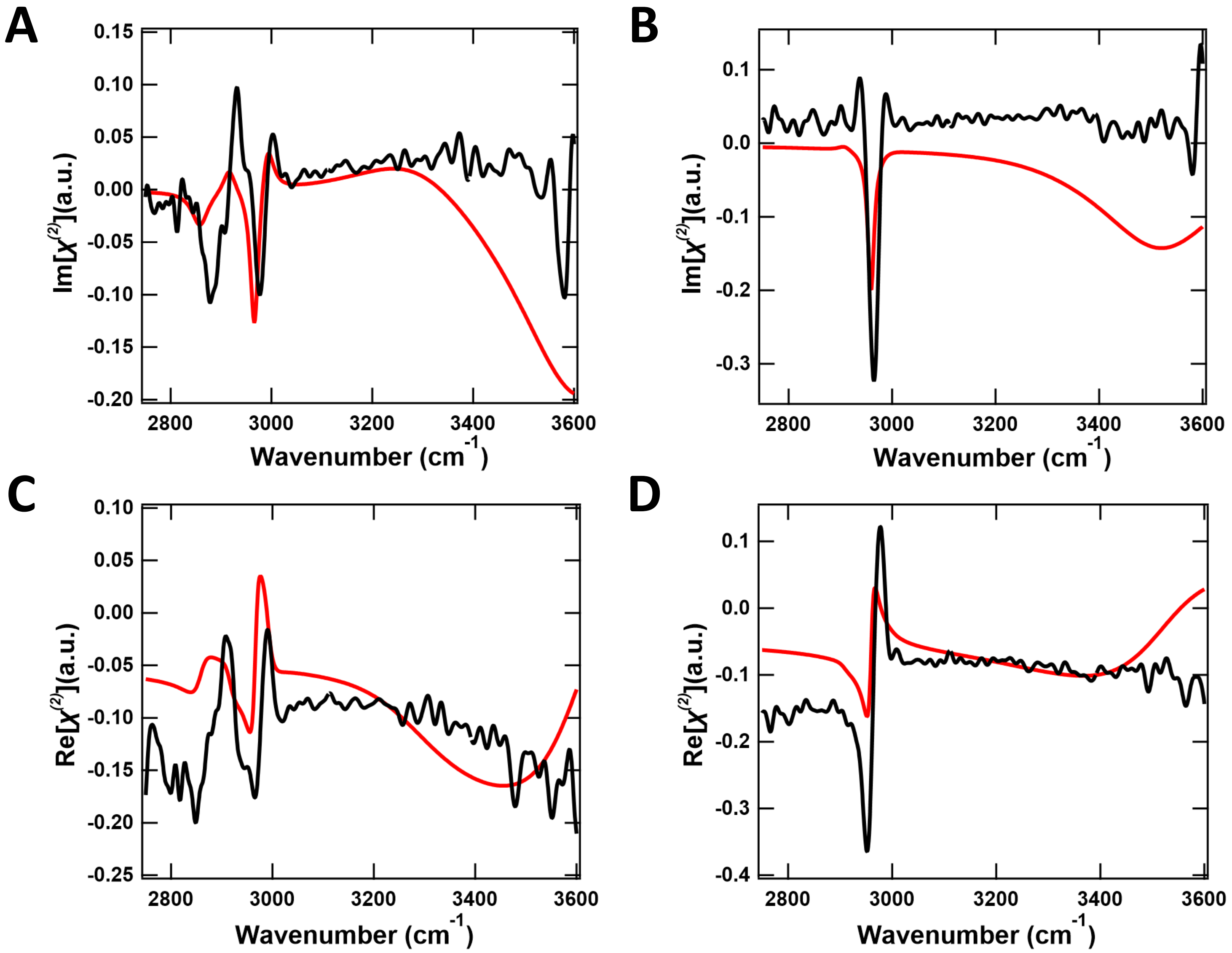

Figure SI-6. Spectra of the imaginary (**A** and **B**) and real (**C** and **D**) parts of simulated (red line) and measured (black line) non-linear susceptibility $\chi^{(2)}$ as a function of wavenumber in SPS polarization for PEO_{11}-PPO_{35}-PEO_{11} (**A** and **C**) and PDMS (**B** and **D**) Langmuir films.

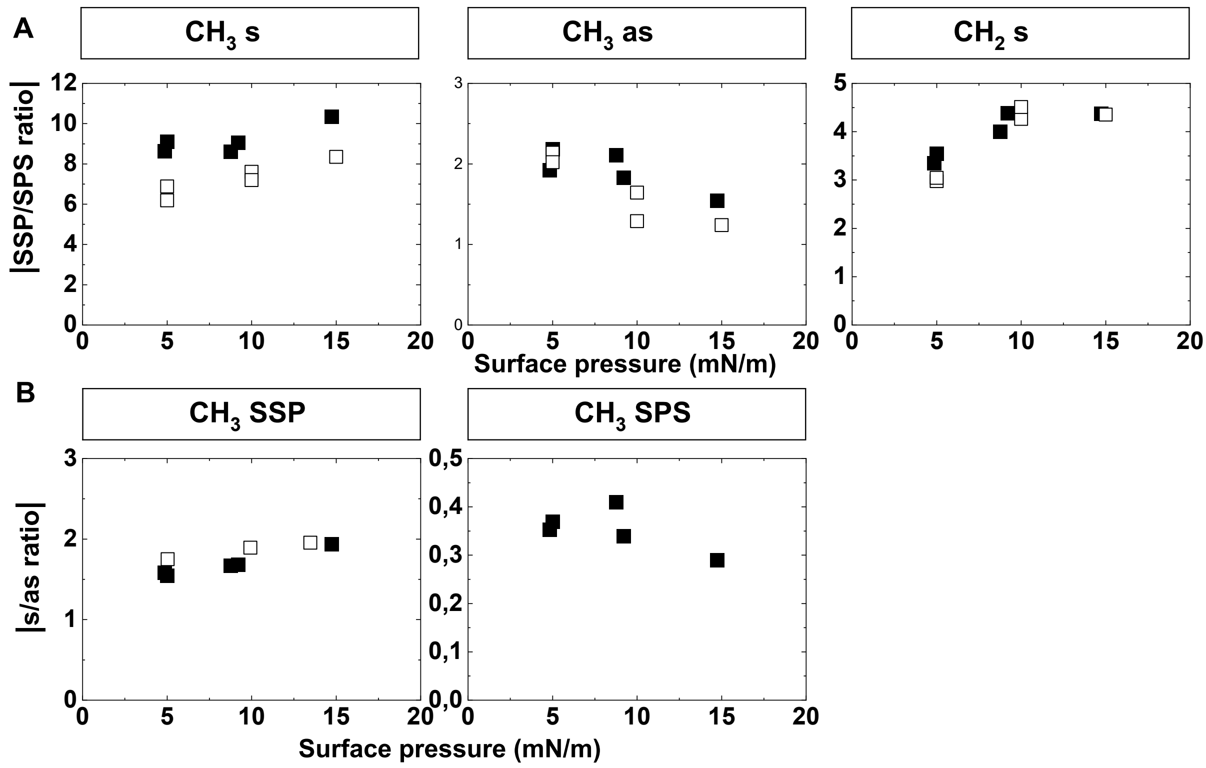

Figure SI-7. Evolution of the absolute value of the SFG amplitude ratio of the CH_3 or CH_2 vibration between **(A)** SSP and SPS polarizations (SSP/SPS ratio) and **(B)** symmetric (s) and asymmetric (as) modes (s/as ratio) in SSP or SPS polarization, as a function of the surface pressure, for the PEO_{11}-PPO_{35}-PEO_{11} Langmuir film. Solid and open squares correspond to two different measurement sets.

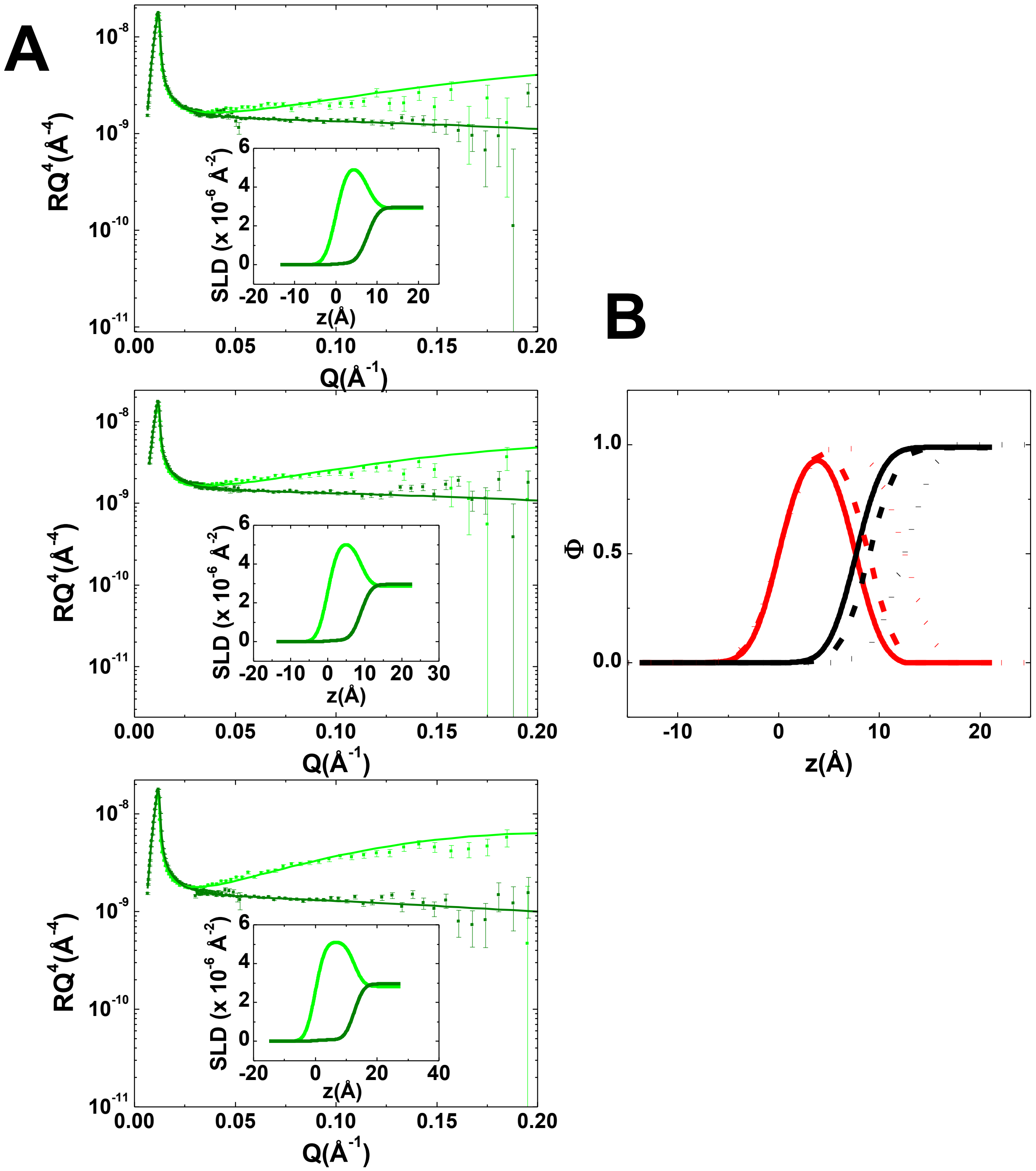

Figure SI-8. A- Fits of the NR curves in RQ^4 representation as a function of wave vector transfer Q ($Å^{-1}$) for pure dPDMS (light green) and PDMS (dark green) Langmuir films at 5 mN/m (top), at the beginning (middle) and end (bottom) of the surface pressure plateau, measured on an H_2O/D_2O subphase with SLD= 3.10^{-6} $Å^{-2}$. The insets show the corresponding SLD profile as a function of depth z. **B-** Volume fraction profiles of PDMS (red) and water (black), Φ_{PDMS} and Φ_{water}, as a function of depth at 5 mN/m (solid line), and at the beginning (A ~ 14.1 $Å^2$, dashed line) and end (A ~ 7.7 $Å^2$, dotted line) of the 9.2 mN/m surface pressure plateau.

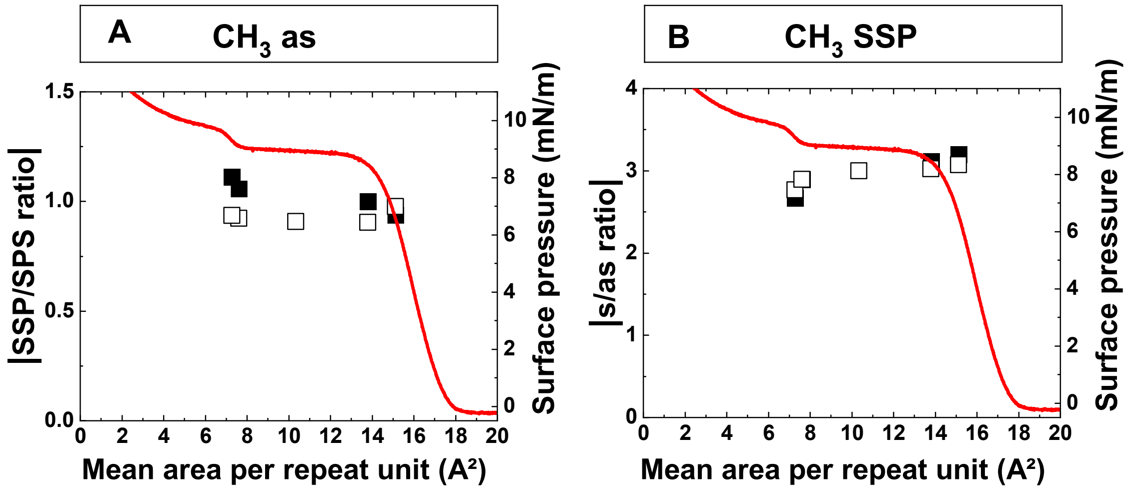

Figure SI-9. Evolution of the absolute value of the SFG amplitude ratio of the CH_3 vibration between **(A)** SSP and SPS polarizations (SSP/SPS ratio) and **(B)** symmetric (s) and asymmetric (as) modes (s/as ratio) in SSP polarization, as a function of the mean area per repeat unit, for the PDMS Langmuir film. The isotherm is shown using a dual-axis format. Solid and open squares represent two independent measurement sets performed on two different films, for each of the SSP and SPS polarizations.

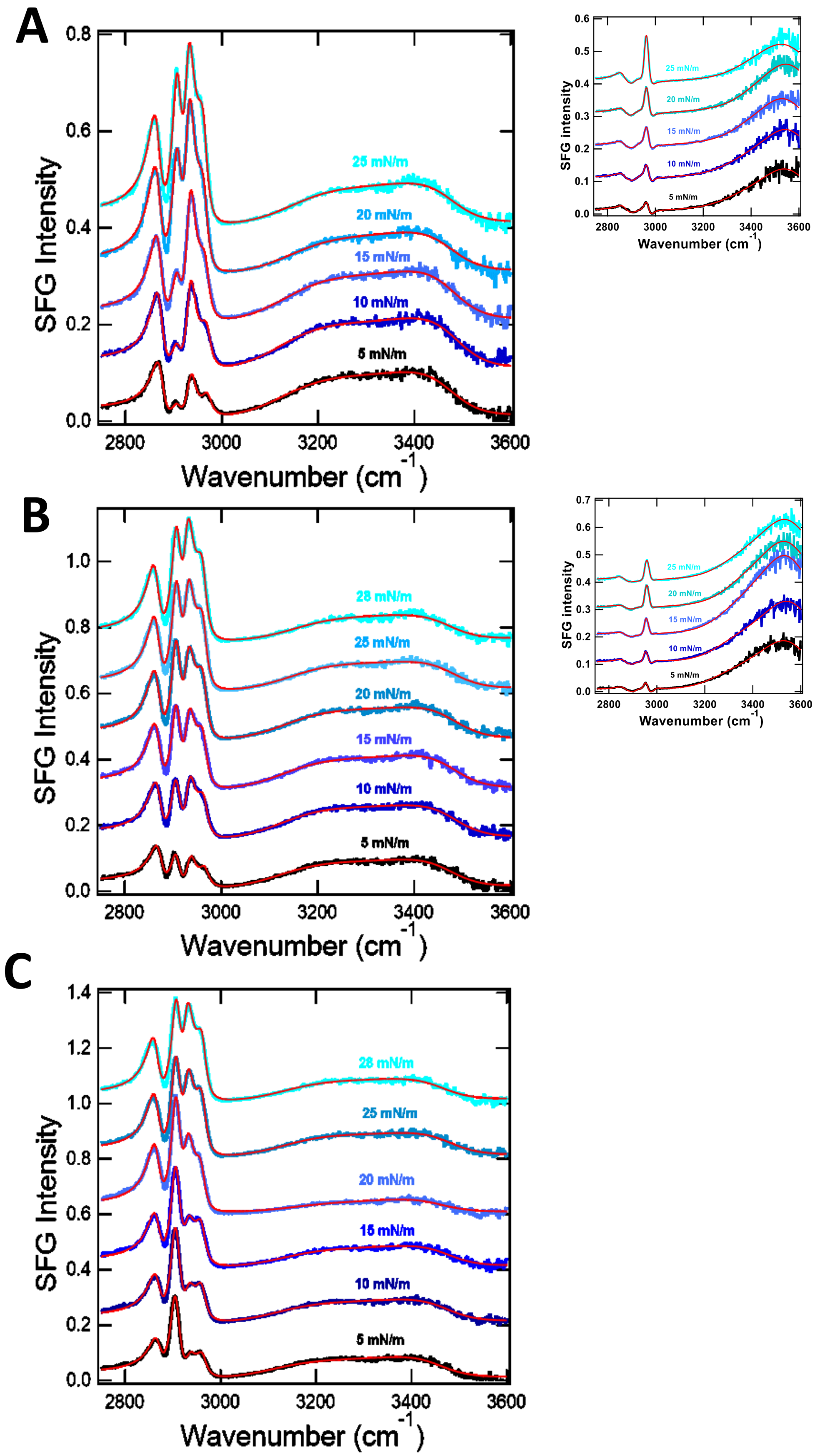
A
B
C
SFG Intensity
Wavenumber (cm-1)
25 mN/m
20 mN/m
15 mN/m
10 mN/m
5 mN/m
28 mN/m
SFG intensity

D

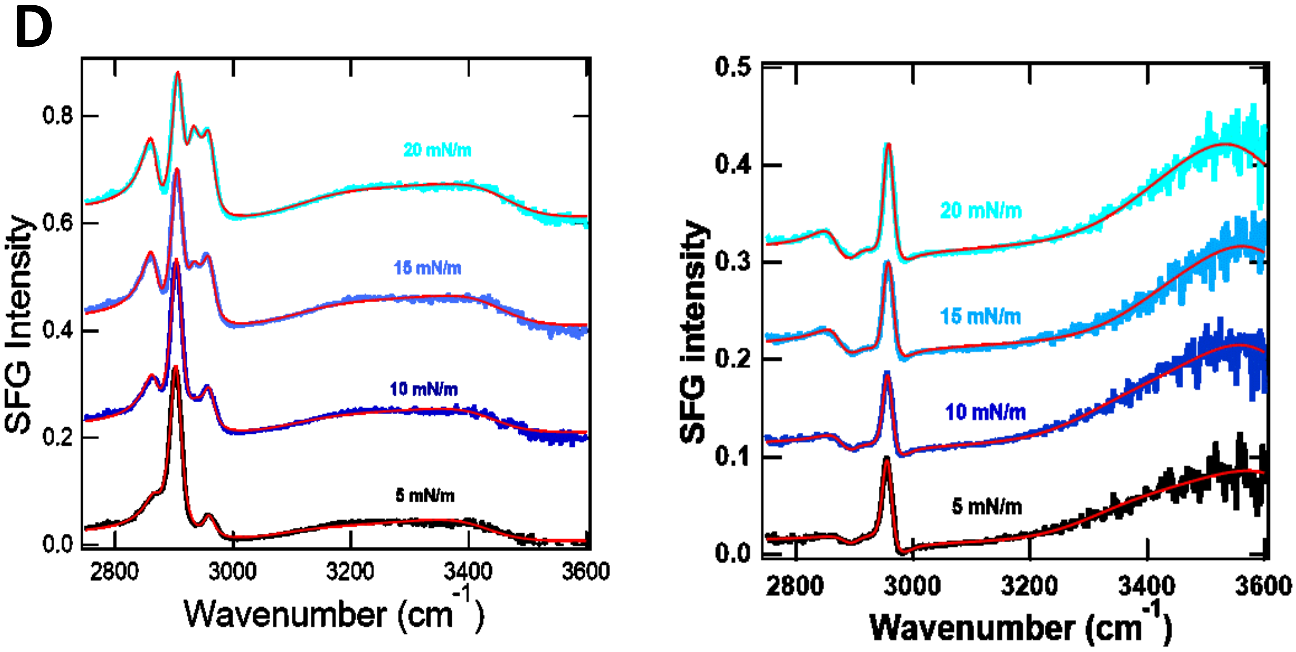

Figure SI-10. SFG spectra in SSP (**left**) and SPS (**right**) polarizations at various surface pressures for PEO_{11}-PPO_{35}-PEO_{11}/PDMS monolayers, with Φ_{PDMS}= 0.28 (**A**), 0.51 (**B**), 0.70 (**C**) and 0.85 (**D**), recorded in the C-H/O-H vibrational region (2750-3600 cm^{-1}). Red lines represent fits using a Lorentzian lineshape model. Spectra are vertically offset for clarity. The SPS spectra for Φ_{PDMS}= 0.70 were not measured.

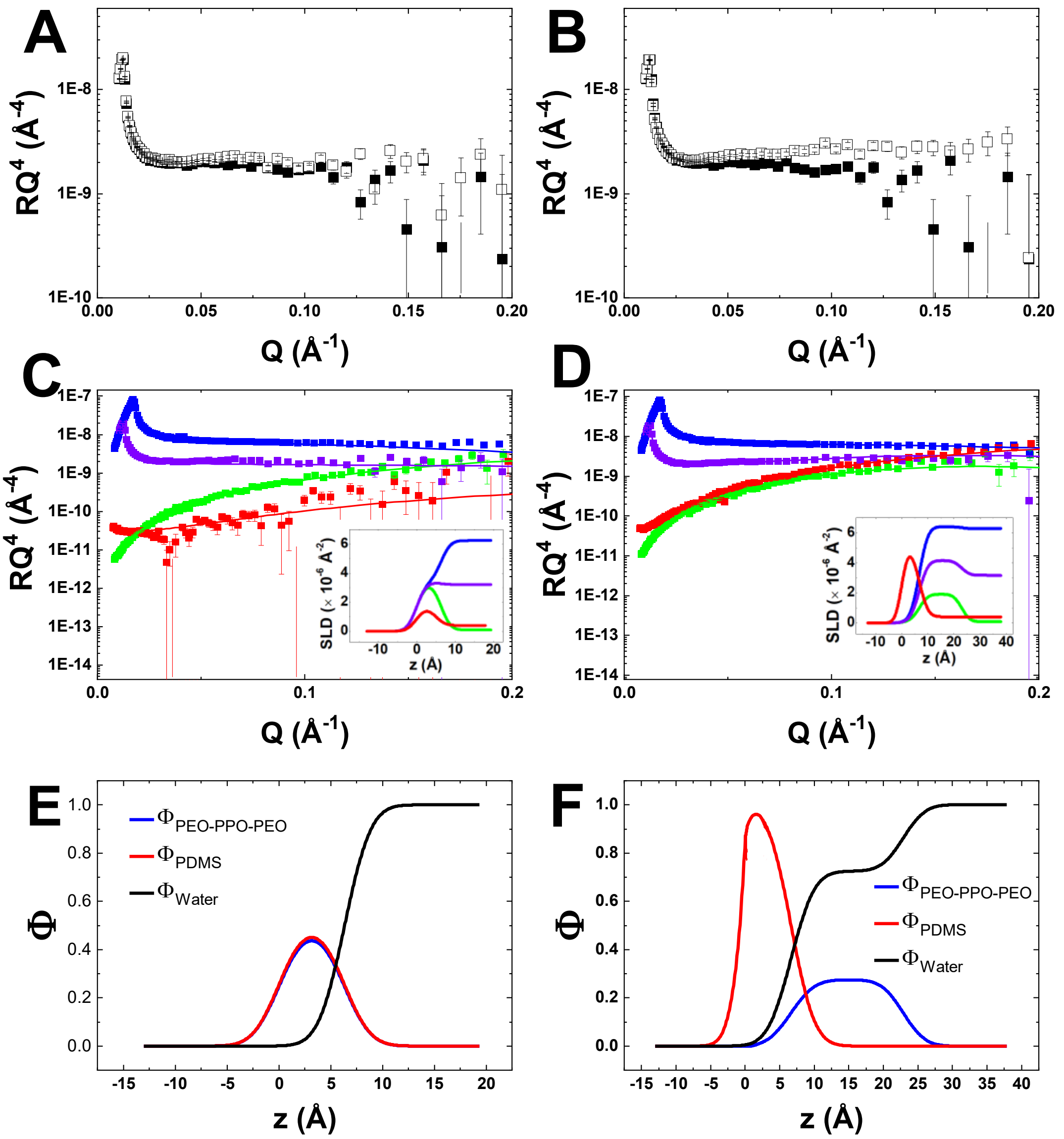

Figure SI-11. NR data for a PEO_{11}-PPO_{35}-PEO_{11}/PDMS film with Φ_{PDMS}= 0.51 at **(A, C, E)** 5 mN/m and **(B, D, F)** 17 mN/m, measured along the green arrow in the phase diagram (Figure 2B). **A and B-** NR curves in RQ^4 representation as a function of wave vector transfer Q ($Å^{-1}$) (open symbols), for $dPEO_{10}$-$dPPO_{34}$-$dPEO_{10}$ /PDMS film on a H_2O/D_2O subphase contrast-matched to the film if homogeneous (SLD = $3.35.10^{-6}$ $Å^{-2}$), compared to the reflectivity of the subphase alone (solid symbols). **C and D-** Fits of the RQ^4 =f(Q) NR curves obtained for $dPEO_{10}$-$dPPO_{34}$-$dPEO_{10}$/PDMS film on three H_2O/D_2O subphases: contrast-matched to PDMS (green), to $dPEO_{10}$-$dPPO_{34}$-$dPEO_{10}$ (blue), to the mixed film if homogeneous (purple), and for PEO_{11}-PPO_{35}-PEO_{11}/dPDMS film on a subphase contrast-matched to PEO_{11}-PPO_{35}-PEO_{11} (red). Insets show the corresponding SLD profiles as a function of depth z. **E and F-** Volume fraction profiles of PEO_{11}-PPO_{35}-PEO_{11} (blue), PDMS (red), and water (black), $\Phi_{PEO_{11}\text{-}PPO_{35}\text{-}PEO_{11}}$, Φ_{PDMS}, and Φ_{water}, as a function of depth.

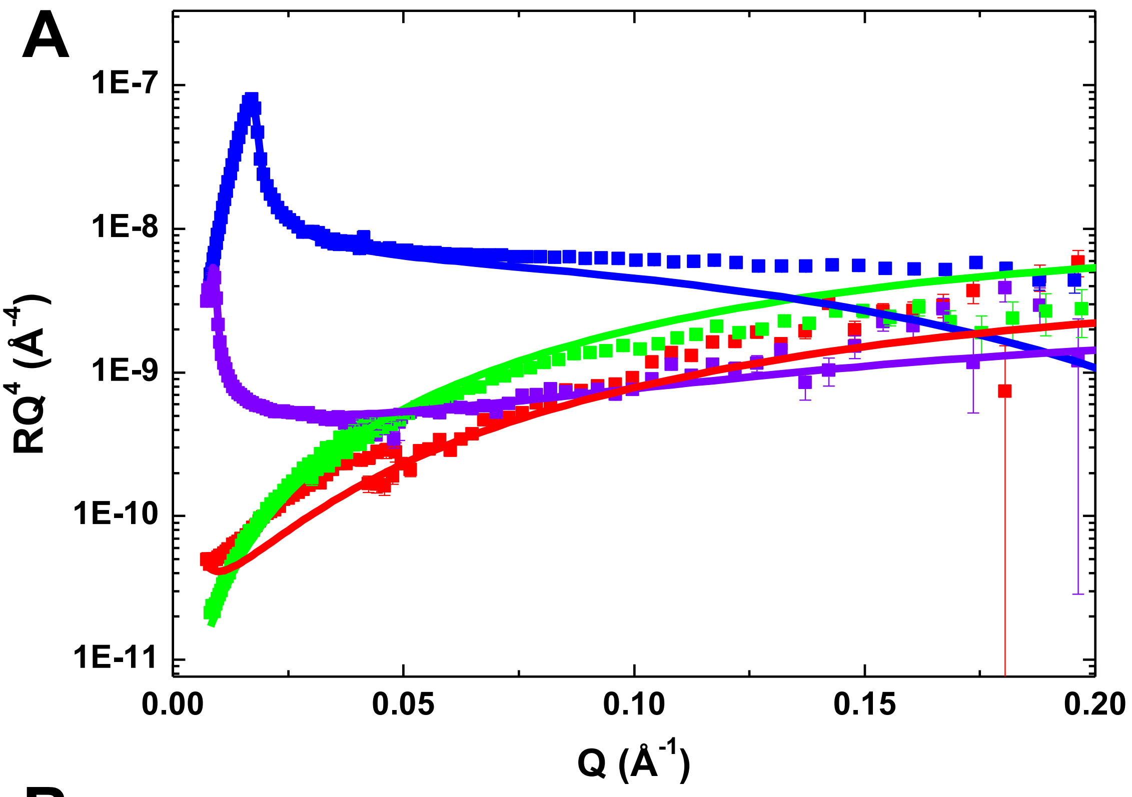

B

Vertical structure	Thickness (Å) / Water fraction	σ_{lower} (Å)	σ_{upper} (Å)
Bilayer:			
PEO-PPO-PEO upper layer	5.7/ 0%	5.0	2.0
PDMS bottom layer	4.4 / 0%	2.0	5.0

Figure SI-12. A- Fits of the RQ^4 =f(Q) NR curves presented in Figure 5D of the main text for a PEO_{11}-PPO_{35}-PEO_{11}/PDMS Langmuir film with Φ_{PDMS}= 0.28 at 23 mN/m,using a model assuming a pure PEO_{11}-PPO_{35}-PEO_{11} layer on top of a pure PDMS layer. **B-** Table summarizing the fitting parameters.

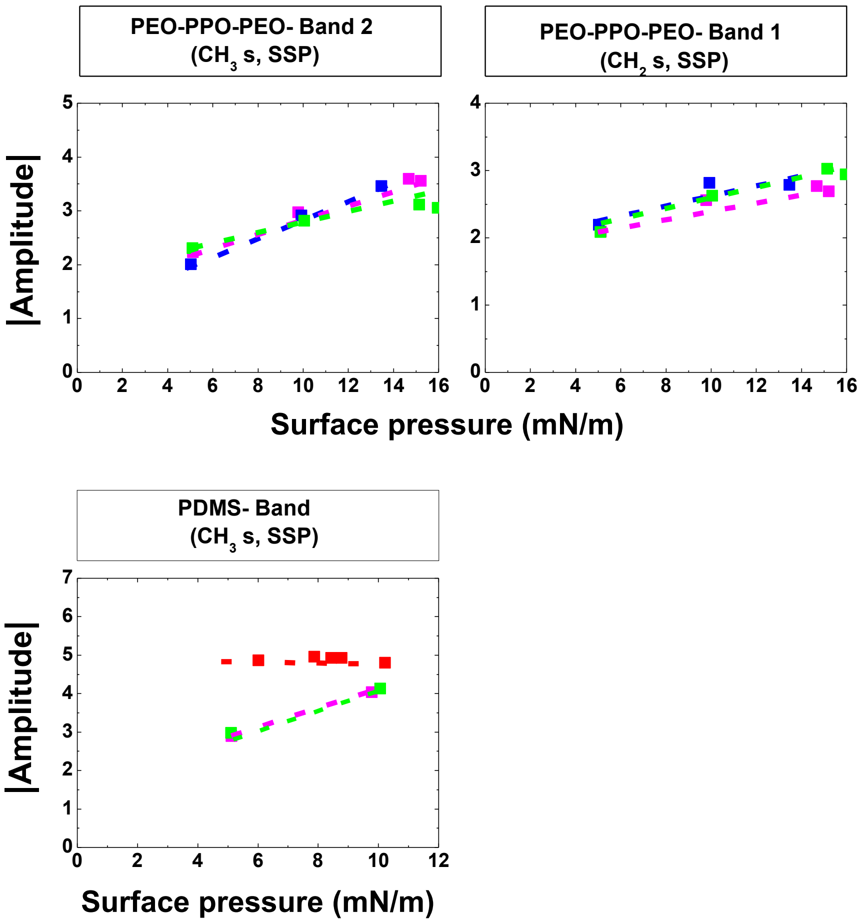

Figure SI-13. Evolution of the absolute value of the normalized amplitude of characteristic SFG bands as a function of surface pressure, measured in SSP polarization (see Table 3), for PEO_{11}-PPO_{35}-PEO_{11}/PDMS blends with Φ_{PDMS}= 0.28 (magenta) and 0.51 (green), compared to the corresponding pure films – PEO_{11}-PPO_{35}-PEO_{11} (blue) or PDMS (red) – depending on the origin of the band. The normalization procedure used to correct the amplitudes for the relative amount of each polymer at the interface is described in Section II.5 of the Experimental part. Dashed lines are included as visual guides.

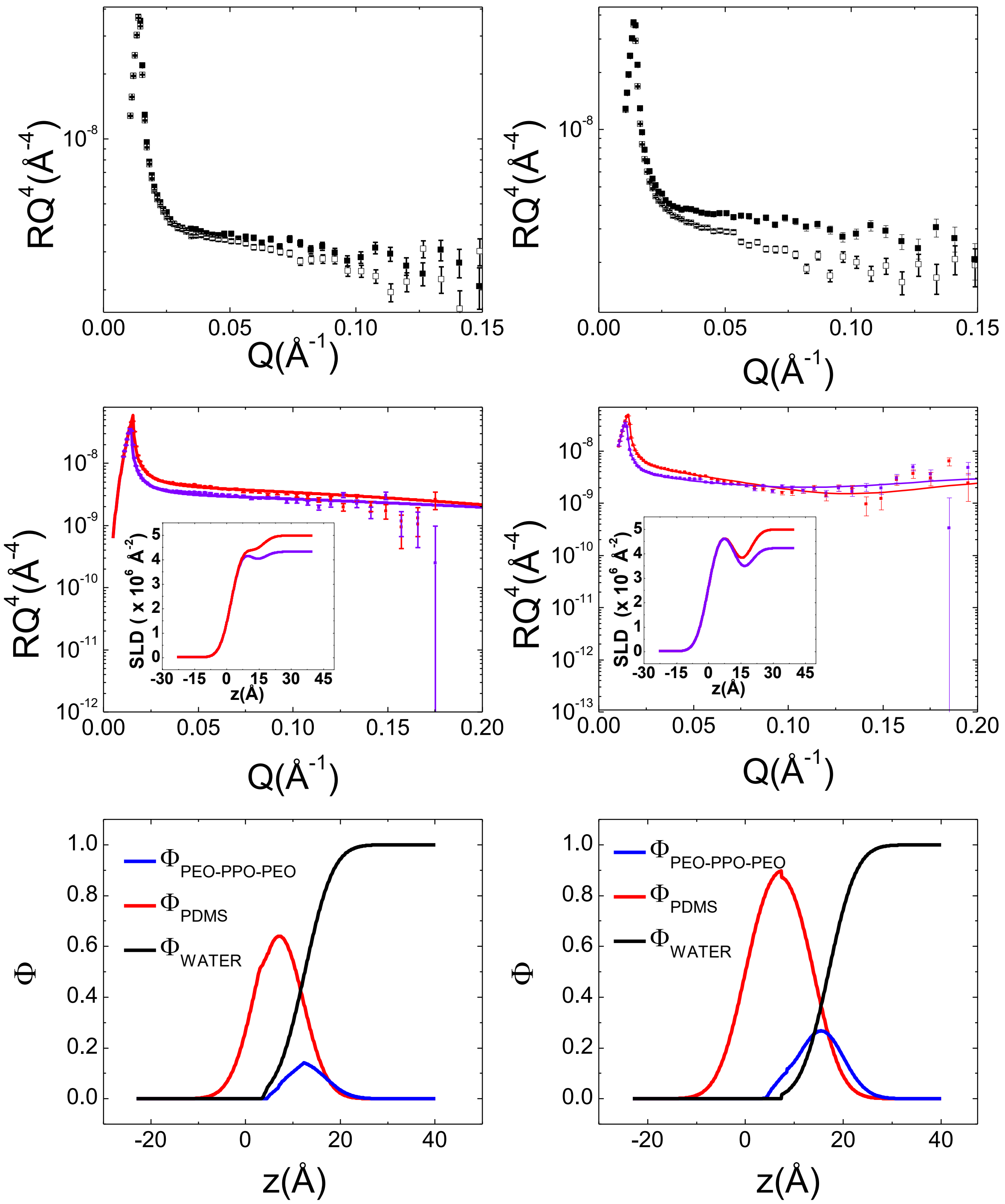

Figure SI-14. NR data for a PEO_{11}-PPO_{35}-PEO_{11}/dPDMS film with Φ_{PDMS}= 0.85 at **(A, C, E)** 5 mN/m and **(B, D, F)** 15 mN/m, measured along the black arrow in the phase diagram (Figure 2). **A and B-** NR curves in RQ^4 representation as a function of wave vector transfer Q ($Å^{-1}$) (open symbols), measured on an H_2O/D_2O subphase contrast-matched to the film if homogeneous (SLD =4.45.10^{-6} $Å^{-2}$) compared to the reflectivity of the subphase alone (solid symbols). **C and D-** Fits of the RQ^4 =f(Q) NR curves obtained using two different H_2O/D_2O subphases: contrast-matched to dPDMS (red), and to the mixed film if homogeneous (purple). Insets show the corresponding SLD profiles as a function of depth z. **E and F-** Volume fraction profiles of PEO_{11}-PPO_{35}-PEO_{11} (blue), PDMS (red), and water (black), $\Phi_{PEO_{11}\text{-}PPO_{35}\text{-}PEO_{11}}$, Φ_{PDMS} and Φ_{water}, as a function of depth.

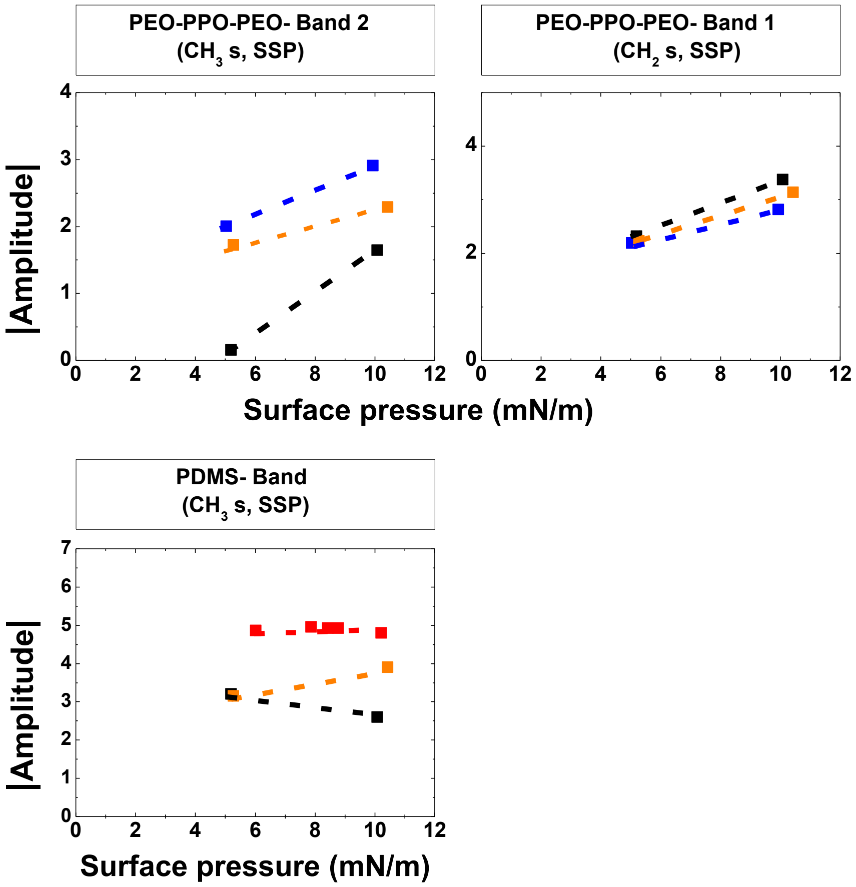

Figure SI-15. Evolution of the normalized amplitude of characteristic SFG bands as a function of surface pressure, measured in SSP polarization (Table 3), for PEO_{11}-PPO_{35}-PEO_{11}/PDMS blends with Φ_{PDMS}= 0.70 (orange) and 0.85 (black), compared to the corresponding pure films – PEO_{11}-PPO_{35}-PEO_{11} (blue) or PDMS (red) – depending on the origin of the band. The normalization procedure applied to adjust the amplitudes for the relative interfacial amount of each polymer is detailed in Section II.5 of the Experimental part. Dashed lines are included as visual guides.
